# Human amygdala involvement in Alzheimer's disease revealed by stereological and dia‐PASEF analysis

**DOI:** 10.1111/bpa.13180

**Published:** 2023-06-18

**Authors:** Melania Gonzalez‐Rodriguez, Sandra Villar‐Conde, Veronica Astillero‐Lopez, Patricia Villanueva‐Anguita, Isabel Ubeda‐Banon, Alicia Flores‐Cuadrado, Alino Martinez‐Marcos, Daniel Saiz‐Sanchez

**Affiliations:** ^1^ Neuroplasticity and Neurodegeneration Laboratory, CRIB, Ciudad Real Medical School University of Castilla‐La Mancha Ciudad Real Spain

**Keywords:** antioxidant protein 2 (AOP2), BM88 antigen (BM88), calpactin II, calpactin‐1 heavy chain (CAL1H), centaurin‐alpha‐1 (CENTA1), endonexin II (ENX2), nuclear chloride ion channel 27 (NCC27)

## Abstract

Alzheimer's disease (AD) is characterized by the accumulation of pathological amyloid‐β (Aβ) and Tau proteins. According to the prion‐like hypothesis, both proteins can seed and disseminate through brain regions through neural connections and glial cells. The amygdaloid complex (AC) is involved early in the disease, and its widespread connections with other brain regions indicate that it is a hub for propagating pathology. To characterize changes in the AC as well as the involvement of neuronal and glial cells in AD, a combined stereological and proteomic analysis was performed in non‐Alzheimer's disease and AD human samples. The synaptic alterations identified by proteomic data analysis could be related to the volume reduction observed in AD by the Cavalieri probe without neuronal loss. The pathological markers appeared in a gradient pattern with the medial region (cortical nucleus, Co) being more affected than lateral regions, suggesting the relevance of connections in the distribution of the pathology among different brain regions. Generalized astrogliosis was observed in every AC nucleus, likely related to deposits of pathological proteins. Astrocytes might mediate phagocytic microglial activation, whereas microglia might play a dual role since protective and toxic phenotypes have been described. These results highlight the potential participation of the amygdala in the disease spreading from/to olfactory areas, the temporal lobe and beyond. Proteomic data are available via ProteomeXchange with identifier PXD038322.

## Graphical abstract

A combined stereological and proteomic analysis revealed amygdala volume reduction linked to synaptic dysfunction in Alzheimer''s disease samples. The pathological markers gradient suggested the relevance of connections in the distribution of the pathology among different brain regions. Extended astrogliosis, likely as response to pathologic deposits, could mediate phagocytic microglial activation. Microglia might play a dual role since protective and toxic phenotypes have been described. In conclusion, amygdala might promote the spreading of pathology from/to olfactory areas, the temporal lobe and beyond.

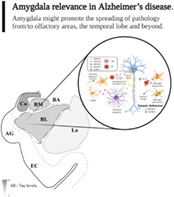

## 1 INTRODUCTION

Alzheimer's disease (AD) is characterized by executive dysfunction and memory impairment [1], with underlying accumulation of extracellular amyloid‐β (Aβ) and intracellular hyperphosphorylated Tau proteins. These two markers form aggregates in a predictable and sequential manner in the different brain regions established as Thal phases [2] and Braak stages [3, 4], respectively. According to the prion‐like hypothesis, both pathological markers can spread from cell to cell throughout brain regions [5, 6]. This premise is in consonance with Braak sequence stages since the affected areas are interconnected [7]. Nevertheless, growing evidence indicates that multiple pathological substrates could be linked to mild cognitive impairment and Alzheimer's clinical syndrome [8, 9]. Recently, limbic‐predominant age‐related TDP‐43 encephalopathy (LATE) has been described as new disease entity characterized by TDP‐43 proteinopathy and Alzheimer's type dementia, being the amygdala involved from early stages [10, 11]. In this sense, the amygdala constitutes a key hub that may contribute to the spread of pathologic molecules because of its vast connectivity with other brain regions [12].

Amygdala atrophy has been described in early stages of the disease [13], and it could be related to certain preclinical symptoms, such as olfactory deficits [14, 15] and/or emotional dysfunctions [16, 17, 18]. Moreover, amygdaloid complex (AC) volume reduction measured with magnetic resonance imaging (MRI) has been proposed as a diagnostic criterion for Alzheimer's disease (AD) [19]. A few histological studies have also confirmed amygdala atrophy [20] accompanied by neuronal and glial loss [21, 22]. However, neither neural nor glial‐specific markers have been employed. Furthermore, the diversity of nomenclature used to identify amygdaloid nuclei together with the lack of consistency in the studied nuclei make it difficult to understand how pathology can affect the AC.

Evidence for glial participation in Aβ and Tau aggregation [23] and propagation [24] has been increasing in recent decades, with special relevance of astrocyte involvement in Tau propagation [25]. Nonetheless, a dual role of glial cells has been postulated since glial‐mediated inflammation might cause damage (propagation) and beneficial effects (pathology clearance) in AD [26]. In this sense, multiple proteomic approaches are now booming with the aim of finding markers of interest. Unfortunately, proteomic analyses in the human amygdala are scarce; either limited to the study of healthy individuals [27] or focused on Aβ extracted from AD samples [28]. However, studies of complete AC in AD associated with the different cell populations are lacking.

Accordingly, the present study includes stereological quantification of volume, cellular populations, and pathology estimations in the AC. In addition, dia‐PASEF analysis of non‐Alzheimer's disease (non‐AD) and AD human amygdala samples was carried out. The aim was to characterize the involvement of neurons, microglia, and astrocytes in the amygdala in AD and to identify markers associated with the different cell populations.

## 2 MATERIALS AND METHODS

### 2.1 Human samples

Human brain samples and data were provided by *Institut d'Investigacions Biomèdiques August Pi i Sunyer*, *Biobanco en Red de la Región de Murcia*, *Biobanco de Tejidos de la Fundación CIEN*, *Biobanco del Principado de Asturias* and *Biobanco Navarrabiomed* (registration numbers: B.0000575, B.0000859, B.0000741, B.0000827, and B.0000735, respectively) integrated in the Spanish National Biobanks Network. The samples were processed following standard operating procedures with the appropriate approval of the Ethical and Scientific Committees. These protocols included obtaining written consent from the donors. All the experimental procedures carried out in the UCAI facilities of the Ciudad Real Medical School were approved by the Ethical Committee of Clinical Research of Ciudad Real University Hospital (SAF2016‐75768‐R and PID2019‐108659RB‐I00).

A total of 36 cases were selected for the study (Table 1): 18 cases were diagnosed as AD, and 18 cases were classified as non‐AD. Formalin‐fixed samples were employed for immunohistochemistry and stereological quantifications (*N* = 20, AD *n* = 10, non‐AD *n* = 10). Fresh‐frozen samples were used for dia‐PASEF analysis (*N* = 16, AD n = 8, non‐AD *n* = 8).

**TABLE 1:** Human samples.

Case	Sex	Age (y)	PMD (h)	Brain weight (g)	Cause of death	Braak stage	Braak syn	TDP‐43	Treatment
AD cases (*N* = 18)									
1	M	88	7:00	1150	Sepsis	V	0	Negative	Formalin fixed
2	F	92	4:00	1000	Respiratory insufficiency	V	0	Positive	Formalin fixed
3	F	62	9:00	900	Cardiorespiratory arrest	V	NA	Negative	Formalin fixed
4	M	59	6:00	1100	Cardiorespiratory arrest	VI	NA	Negative	Formalin fixed
5	F	91	7:00	NA	Pulmonary thromboembolism	V	NA	Negative	Formalin fixed
6	F	74	4:00	1042	Cardiorespiratory arrest	V	NA	Negative	Formalin fixed
7	M	77	6:00	1060	Acute respiratory infection	VI	0	Negative	Formalin fixed
8	F	71	10:00	1006	NA	V	0	Negative	Formalin fixed
9	F	68	NA	1100	Gastric carcinoma	VI	0	Negative	Formalin fixed
10	F	89	NA	910	NA	V	0	NA	Formalin fixed
11	F	91	8:00	1080	Respiratory insufficiency	V	0	Negative	Fresh‐frozen
12	M	78	5:00	1260	Multiorganic arrest	V	0	Negative	Fresh‐frozen
13	M	67	4:05	1100	Acute respiratory insufficiency	VI	0	Positive	Fresh‐frozen
14	M	85	3:15	1130	Upper gastrointestinal bleeding	VI	0	Positive	Fresh‐frozen
15	F	67	4:15	1160	Bronchoaspirative pneumonia	VI	0	Negative	Fresh‐frozen
16	M	69	2:25	900	Multiorganic arrest	VI	5	Negative	Fresh‐frozen
17	F	76	11:10	900	Respiratory insufficiency	VI	0	Negative	Fresh‐frozen
18	F	85	5:00	960	Respiratory insufficiency	V	0	Negative	Fresh‐frozen

Non‐AD cases (*N* = 18)									
19	M	56	19:00	1400	Cardiorespiratory arrest	I	NA	Negative	Formalin fixed
20	M	84	3:00	1400	Cardiorespiratory arrest	‐	NA	Negative	Formalin fixed
21	M	74	7:00	1336	Tumor of unknown origin	I	0	Negative	Formalin fixed
22	M	88	3:00	1285	NA	II	0	NA	Formalin fixed
23	F	58	5:00	944	Pneumonia	‐	0	Negative	Formalin fixed
24	F	59	4:00	1200	Respiratory insufficiency	‐	NA	Negative	Formalin fixed
25	M	63	2:00	1400	Cardiorespiratory arrest	I	NA	Negative	Formalin fixed
26	F	62	2:00	1050	Sepsis	‐	NA	Negative	Formalin fixed
27	F	83	4:00	1152	NA	II	0	Negative	Formalin fixed
28	M	86	7:00	965	Respiratory insufficiency	II	NA	Negative	Formalin fixed
29	F	71	7:08	975	Cardiorespiratory arrest	‐	0	Negative	Fresh‐frozen
30	M	68	4:00	1220	Cardiorespiratory arrest	‐	0	Negative	Fresh‐frozen
31	M	68	4:10	1350	Sepsis	‐	0	Negative	Fresh‐frozen
32	M	77	10:31	1300	Bronchoaspiration	‐	0	Negative	Fresh‐frozen
33	M	72	2:55	1340	Systemic vascular pathology	‐	NA	Negative	Fresh‐frozen
34	F	68	16:30	1076	Refractory asystolia	‐	NA	Negative	Fresh‐frozen
35	M	81	5:00	1309	Respiratory pathology	‐	NA	Negative	Fresh‐frozen
36	M	72	9:00	1407	‐	‐	NA	Negative	Fresh‐frozen

*Note*: Detailed information about the samples employed in the study, including sex, age, postmortem delay, brain weight, cause of death, Braak stage, and treatment of the sample.

Abbreviations: F, female; M, male; NA, not available; PMD, postmortem delay; y, years.

Formalin‐fixed samples from different tissue banks were postfixed in fresh phosphate‐buffered 4% paraformaldehyde for 45 days. For cryoprotection, blocks were immersed for 48 h in a phosphate buffered (PB) solution of 2% dimethyl sulfoxide (DMSO) and 10% glycerol and for 48 h in a PB solution of 2% DMSO and 20% glycerol. A freezing sliding microtome was used to obtain 50‐μm‐thick coronal sections. Thirteen series were obtained for each block, and the distance between sections was 650 μm. The first series was used for Nissl staining. The remaining series were stored in 24‐well plates at −20°C in 30% ethylene glycol and 20% glycerol in 0.1 M PB (pH 7.4).

Frozen samples were homogenized following previously described procedures [29, 30, 31]. Briefly, tissue was homogenized in 0.4 mL of RIPA buffer (50 mM Tris–HCl pH 7.4, 150 mM NaCl, 0.1% Triton X‐100, 0.1% SDS, and 0.5% Na‐deoxycholate) containing a protease inhibitor cocktail (Sigma–Aldrich) and incubated for 2 h at 4°C. Protein extraction was performed by centrifugation at 12,000*g* for 5 min at 4°C, and the supernatant was collected.

### 2.2 Immunohistochemistry

Tissue epitopes were unmasked by boiling the tissue under pressure for 2 min in citrate buffer. The sections were immersed in formic acid for 3 min and rinsed in phosphate‐buffered saline (PBS). Endogenous peroxidase activity was inhibited by incubation in 1% H_2_O_2_ in PBS for 20 min. The sections were preincubated for 1 h (microtubule‐associated protein 2 [MAP2] and allograft inflammatory factor 1 [Iba‐1]) or 2 h (glial fibrillary acidic protein [GFAP], Tau and Aβ) with blocking buffer and overnight at 4°C with primary antibodies (MAP2, Iba‐1, GFAP, Tau, and Aβ) (for details, see Online Resource 1). The sections were then incubated in biotinylated anti‐rabbit secondary antibody (1:200; Vector Laboratories) for 2 h at room temperature and in avidin–biotin complex (ABC Standard; Vector Laboratories) and reacted with 0.025% 3.3′‐diaminobenzidine and 0.1% H_2_O_2_. The sections were mounted, counterstained with Nissl, dried, dehydrated, and coverslipped with DPX (Sigma–Aldrich).

### 2.3 Stereological quantifications

Human amygdala volume and neuronal, microglial and astroglial cell populations were quantified using a Zeiss Axio Imager M.2 microscope coupled to stereological software (StereoInvestigator, MBF Bioscience®). The amygdaloid nuclei were delimited with a 1× objective (Zeiss Plan‐Neofluar 1×/0.025, Ref. 420300‐9900), and quantification was performed under a 63× objective (Zeiss Plan‐Apochromat 63×/1,4 oil DIC, Ref. 420782‐9900).

Volume estimation was carried out using the Cavalieri estimator probe. The number of MAP2‐, Iba‐1‐, and GFAP‐expressing cells was quantified using the optical fractionator method. The dissector height (*Z*) was 9 μm, and the guard zones were 2 μm. The Tau‐ and Aβ‐positive areas were assessed with the area fraction fractionator (AFF) method under 40× (Zeiss Plan‐APOCHROMAT 40×/0.95, Ref. 420660‐9970) and 20× objectives (Zeiss Plan‐APOCHROMAT 20×/0.8, Ref. 420650‐9901), respectively.

### 2.4 Statistical analysis

For stereological quantifications, the normality of the data was assessed using the Shapiro Wilk test. The data are expressed as the mean ± SEM. For normal data, mean values were compared using either *t* tests or one‐way ANOVA, and the Mann–Whitney *U* test was used for non‐normal data. *F* tests were carried out to compare variables, and in the case of differences between variables, *t* tests with Welch's correction were performed. The ROUT method was employed for outlier identification. No data were removed for the analysis. A significance level of *α* = 0.05 was used. Statistical analyses were performed with the GraphPad Prism 8.0.2 software.

### 2.5 dia‐PASEF proteomic analysis

#### 2.5.1 Sample preparation

Samples were precipitated using methanol/chloroform and resuspended in 100 μL of RapiGest SF (Waters). Total protein concentration was measured using the Qubit fluorimetric protein assay (Thermo Fisher Scientific). Twenty‐five micrograms of protein were digested using the iST kit (PreOmics). Peptides were diluted using LC–MS H_2_O 0.1% (v/v) formic acid to 10 ng/μL. Two hundred nanograms of peptides were loaded onto Evotips (Evosep) for purification. Pierce HeLa tryptic Digest Standard (Thermo Fisher Scientific) was also loaded for quality control.

#### 2.5.2 LC–MS/MS

Liquid chromatography–tandem mass spectrometry (LC–MS/MS) was carried out using an Evosep One LC system (Evosep) coupled to a TIMS Q‐TOF instrument (timsTOF Pro, Bruker Daltonics) via a nanoelectrospray ion source (Captive Spray Source, Bruker Daltonics). An MS/MS peptide library was built from the peptides and proteins identified using data‐dependent acquisition (DDA) parallel accumulation‐serial fragmentation (PASEF) analyses of the samples. Each sample was analyzed using the same liquid chromatography–mass spectrometry (LC–MS) system and gradient as used for the previous DDA runs but using data independent acquisition (DIA) (for details, see Online Resource 2).

#### 2.5.3 Protein identification

Peptide identification was performed using MSFragger. Databases of *H. sapiens* protein sequences (UP000005640) from UniProt (reviewed sequences only; Apr 2021) and common contaminating proteins, which contained 20,382 total sequences, were used. Inverted protein sequences were added to the original databases. The initial mass tolerance was set at 20 ppm for precursor and fragment ions. Trypsin was set as described above with a maximum of two missed cleavages. Methionine oxidation and N‐terminal acetylation were established as variable modifications, and carbamidomethylation was established as a fixed modification. Peptide lengths of 7–50 amino acids and peptide masses of 500–5000 Da were set. A maximum of three variable modifications per peptide was set. PeptideProphet was used to calculate the probability of correct identification of peptides for spectrum matching and to assemble peptides into proteins. Philosopher Filter was used to assign each identified peptide as a razor peptide to a single protein or protein group that had the greatest peptide evidence. The false discovery rate (FDR) was set to 1% for peptide spectrum match or ion/peptide and protein identification. EasyPQP was used for aligning peptides to a common indexed retention time scale and peptide ion mobility to that from one of the references runs automatically selected. The final spectral library was filtered at 1% FDR at the peptide and protein levels.

DIA‐NN 1.8 (https://github.com/vdemichev/DiaNN/releases/tag/1.8) was used for diaPASEF analysis and operated with maximum mass tolerances set to 15 ppm. The samples were analyzed with run‐to‐run pairing (match between ranks) enabled. Protein inference in DIA‐NN was configured to use the assembled proteins in the spectral library. Protein. The group column in the DIA‐NN report was used to identify the protein group and PG. MaxLFQ label‐free quantification was used to obtain the normalized amount. The DIA‐NN output was filtered at a q value <1% for precursors and proteins.

The FDR validation was filtered to include only unmodified peptides or peptides with carbamidomethylated cysteines, oxidized methionines, or excised N‐terminal methionines. The library was screened for precursors/proteins with a 2–4 charge range and a 100.0–1700.0 m/z mass range.

LC–MS/MS, protein identification and quantification were carried out at the *Instituto Maimonides de Investigación Biomédica de Córdoba* (IMIBIC) Proteomic Facility. The mass spectrometry proteomics data have been deposited to the ProteomeXchange Consortium via the PRIDE [32] partner repository with the dataset identifier PXD038322.

#### 2.5.4 Proteomic data analysis

Perseus (1.6.15.0) was used to analyze identified proteins. After log2 transformation, data were normalized using the width adjustment method. Proteins with one razor peptide and missing values were removed. An unpaired two‐tailed t test was employed to estimate significant differences. The fold change (FC) cut off was established at 1.5, and a *p* value <0.05 was used to obtain differentially expressed proteins (DEPs). SynGo (dataset version: 20210225) and Metascape [33] were employed for functional analysis of synapses and processes. Lists of proteins that interact with pathological markers (APP and MAPT) were obtained with BioGRID^4.4^ [34]. Proteins expressed preferentially in each cellular type (neurons, microglia, and astrocytes [35]) were compared with DEPs and pathological marker interactomes using Venn diagrams.

#### 2.5.5 Immunofluorescence

To validate proteomic data, tissue epitopes were unmasked, and sections were preincubated for 1 h with blocking buffer and overnight at 4°C with primary antibodies (for details, see Online Resource 1). Subsequently, the sections were incubated with Alexa Fluor 488‐conjugated anti‐rabbit, Alexa Fluor 594‐conjugated anti‐mouse or Alexa Fluor 647‐conjugated anti‐goat antibodies (1:200; Thermo Fisher) for 2 h and then with 0.05% DAPI for 10 min at room temperature. Sections were mounted and coverslipped with PVA‐DABCO.

#### 2.5.6 Confocal analysis

Triple immunofluorescence staining of pathological proteins and proteins identified by dia‐PASEF analysis was analyzed with a Zeiss LSM 800 confocal microscope coupled to the Zen 2.3 software (Oberkochen, Germany). Spatial colocalization was analyzed in high magnification images obtained with a 63× objective (Zeiss Plan‐Apochromat 63×/1.4 Oil DIC M27‐oil, Ref. 420782‐9900‐799).

## 3 RESULTS

### 3.1 Volume reduction in the human amygdala

**FIGURE 1:**
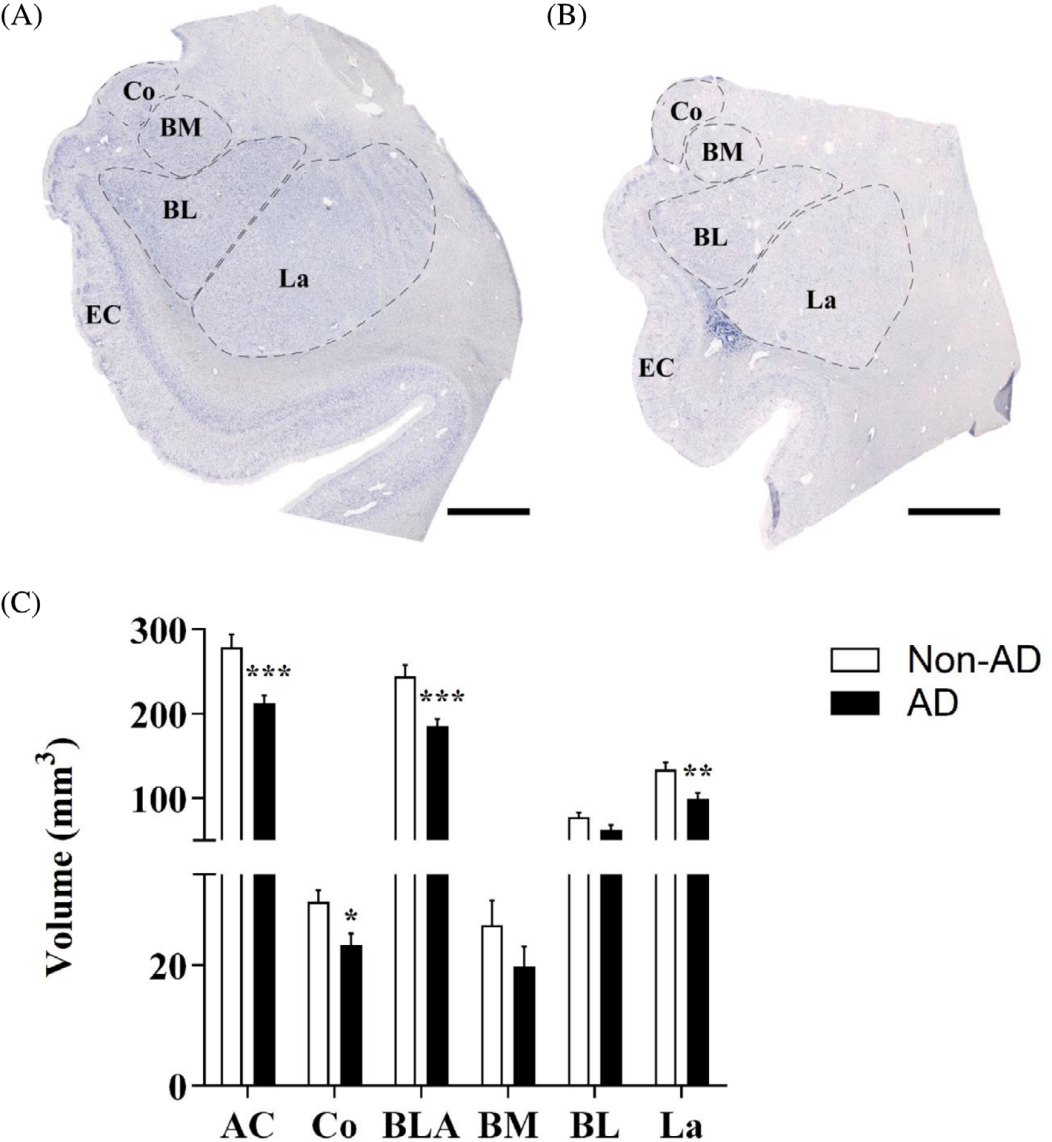
Amygdaloid volume reduction is specific to the Co and BLA, in particular the La. Nissl staining of the non‐AD (A) and AD (B) in the AC with delimitation of the amygdaloid nuclei studied. The global AC volume (C) and volume of the Co and BLA were significantly reduced in AD. In the BLA, volume was reduced specifically in the La (the graphs show the volume mean ± SEM, **p value <0.01, ***p value <0.001). AC, amygdaloid complex (Co, BLA); Co, cortical nucleus; BLA, basolateral complex (BM, BL, La); BM, basomedial nucleus; BL, basolateral nucleus; La, lateral nucleus. Scale bar = 1000 μm.

Nissl staining of human amygdala samples was employed for delimitation and volume estimation of the cortical nucleus (Co) and the basolateral complex (BLA), including its basomedial (BM), basolateral (BL), and lateral (La) nuclei (Figure 1A,B) (for nomenclature used, see [36]; in the present study, the Co plus BLA was referred to as the AC). The Cavalieri probe revealed a volume reduction in the AC (Mann–Whitney *U* = 5.000, *p* value = 0.0002) and particularly in the Co (unpaired *t* test *t*18 = 2.589, *p* value = 0.0185) and BLA (Mann–Whitney *U* = 5.000, *p* value = 0.002). When the different nuclei of the BLA were analyzed, a specific volume reduction in La was observed (unpaired *t* test *t*18 = 3.032, *p* value = 0.0072; Figure 1C; for detailed information on stereological data of volume estimations, see Online Resource 3).

### 3.2 Cell population analysis revealed generalized astrogliosis in the AC in AD

**FIGURE 2:**
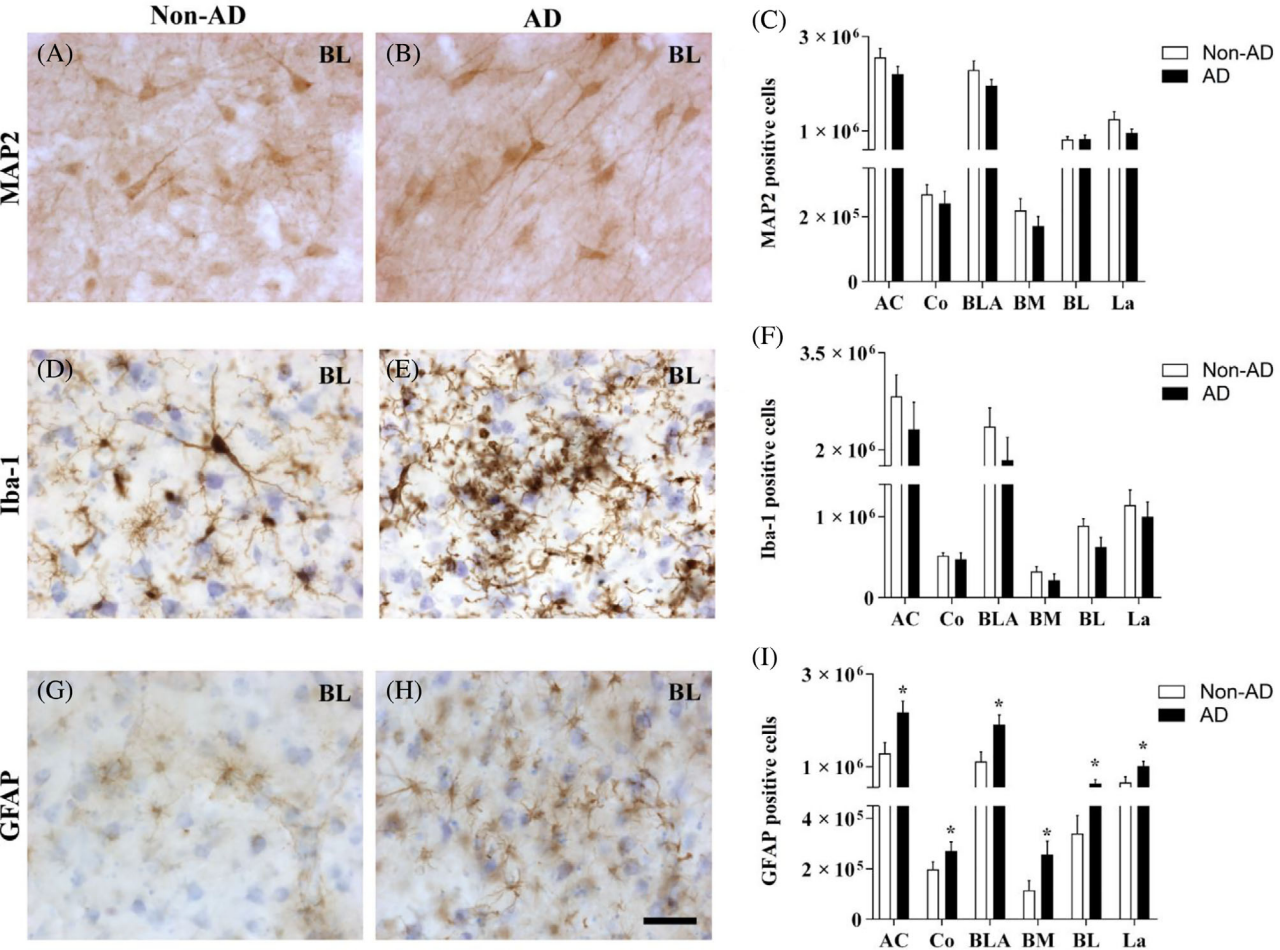
Generalized astrogliosis in the amygdaloid nuclei in AD. Immunohistochemical staining for MAP2 (A,B), Iba‐1 (D,E), and GFAP (G,H) in the BL in non‐AD and AD samples represents neurons, microglia, and astrocytes, respectively. The number of MAP2‐positive cells (C), Iba‐1‐positive cells (F), and GFAP‐positive cells (I) in the global AC and in the different nuclei are shown (the graphs show the mean ± SEM, *p value <0.05). Note that neither the number of neurons nor microglia was altered, and the number of astrocytes was increased in the whole AC. AC, amygdaloid complex (Co, BLA); Co, cortical nucleus; BLA, basolateral complex (BM, BL, La); BM, basomedial nucleus; BL, basolateral nucleus; La, lateral nucleus. Scale bar = 50 μm.

Quantification of MAP2 (Figure 2A,B) and Iba‐1 (Figure 2D,E) positive cells revealed no differences in the number of neurons (Figure 2C) or microglia (Figure 2F). Microglial morphology was largely different in the non‐AD group (Figure 2D) compared with the AD group (Figure 2E), suggesting possible microglial activation in response to pathology. Regarding GFAP quantification (Figure 2G,H), a significant increase in the number of GFAP‐positive cells in the AC (unpaired *t* test *t*18 = 2.673, *p* value = 0.0155) as well as in every analyzed nucleus was reported (Co: Mann–Whitney *U* = 18.00, *p* value = 0.0279; BLA: Mann–Whitney *U* = 17.00, *p* value = 0.0115; BM: Mann–Whitney *U* = 19.00, *p* value = 0.0185; BL: Mann–Whitney *U* = 18.00, *p* value = 0.0147; La: Mann–Whitney *U* = 18.00, *p* value = 0.0147; Figure 2I).

Concerning cell densities, neither neurons nor microglia showed changes (Online Resource 4). However, GFAP‐positive cell density was increased in the AC (unpaired *t* test *t*18 = 4.019, *p* value = 0.0008) and its different nuclei as well (Co: Mann–Whitney *U* = 14.00, *p* value = 0.0101; BLA: unpaired *t* test *t*18 = 3.905, *p* value = 0.001; BM: Mann–Whitney *U* = 7.00, *p* value = 0.0005; BL: unpaired *t* test *t*18 = 3.560, *p* value = 0.0022; La: unpaired *t* test *t*18 = 4.004, *p* value = 0.0008; Online Resource 4; for detailed information on stereological data of MAP2, Iba‐1 and GFAP estimations, see [Link], [Link], and 7, respectively).

### 3.3 Cortical and basal regions are the most affected by pathology in AD

**FIGURE 3:**
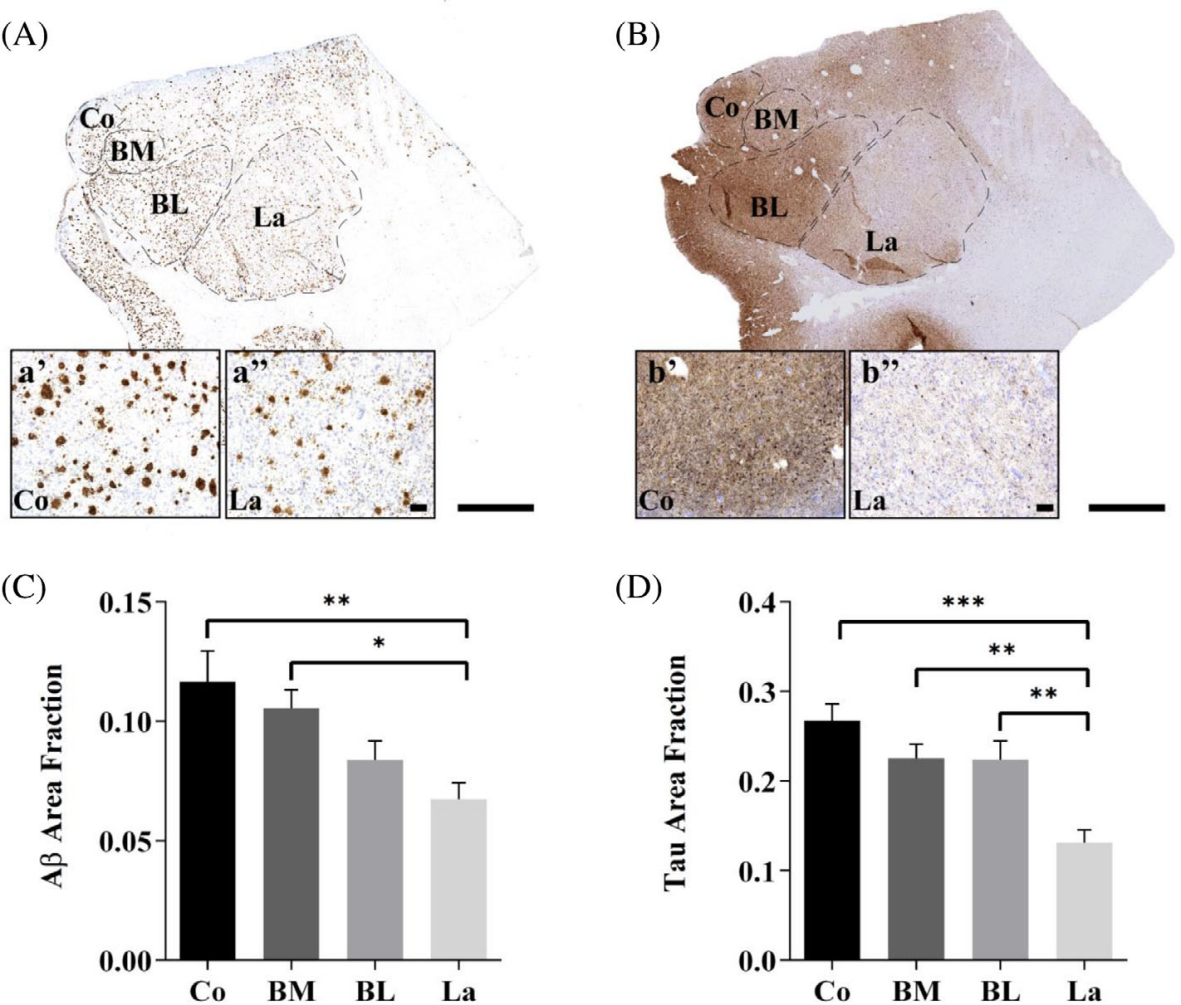
The cortical region is the most affected by pathology in AD. Aβ (A) and Tau (B) immunohistochemical staining of AD samples. Detail of Aβ (A', A'') and Tau (B', B'') staining pattern observed in Co and La, respectively. The area fractions of Aβ (C) and Tau (D) in the global AC and the different nuclei are shown (the graphs show the mean ± SEM, *p value <0.05, **p value <0.01, ***p value <0.001). Note that both Aβ and Tau appeared as a gradient with higher levels in medial (Co) regions than in lateral regions. Co: Cortical nucleus, BM: Basomedial nucleus, BL: Basolateral nucleus, La: Lateral nucleus. Scale bar = 1000 μm in (A,B); and 100 μm in (A',A''; B',B'').

The analysis of the area fraction occupied by pathological markers revealed a strong difference between the cortical and basal regions (BA; corresponding to the BM and BL) compared with La (Figure 3A,B). The area fraction occupied by Aβ was larger in the Co and BM than in the La (Figure 3C, one‐way ANOVA *F* (3, 36) = 5.726, *p* value = 0.0026), and the Tau area fraction was larger in the Co, BM, and BL than in the La (Figure 3D, one‐way ANOVA *F* (3, 36) = 10.74, *p* value <0.0001). Despite the differences in the staining pattern (Figure 3A,B), both Aβ and Tau appeared as a gradient with higher levels in medial (Co) regions (Figure 3A',B') than in lateral regions (Figure 3A'',B''; for detailed information on Aβ and Tau stereological data, see Online Resources 8 and 9, respectively).

In addition, because of the relevance of the amygdala regarding TDP‐43 related pathology, we performed an immunohistochemistry against phosphorylated TDP‐43 (TDP‐43‐P) selecting one of the positive cases provided by the biobanks (Table 1) (Online Resource 10). Interestingly, the staining of TDP‐43‐P was distributed in a similar manner as observed in Aβ and Tau labeling (Online Resource 10A). Whereas numerous intracellular accumulations of TDP‐43‐P were presented in Co (Online Resource 10B), clusters of TDP‐43‐P were common in BM and BL (Online Resource 10C,D, respectively). In contrast, scarce TDP‐43‐P deposits were found in La (Online Resource 10E).

### 3.4 Proteomic analysis revealed synaptic alteration and cellular responses to stress, with potential participation of astroglia and microglia

After restricted conditions of FC > 1.5 and *p* value <0.05 were applied to the 2153 quantified proteins by dia‐PASEF, a total of 178 proteins were considered DEPs in the proteomic analysis. From the 178 DEPs, 108 were considered up‐ and 70 were downregulated in AD (Table 2).

**TABLE 2:** Differentially expressed proteins in AD amygdala.

Protein IDs	Protein names	Genes	Protein description	FC	*p* value
Upregulated proteins					
P05362	ICAM1_HUMAN	*ICAM1*	Intercellular adhesion molecule 1	4.95	0.0001
Q8IV08	PLD3_HUMAN	*PLD3*	5′‐3′ exonuclease PLD3	4.21	0.0496
P16070	CD44_HUMAN	*CD44*	CD44 antigen	3.91	0.0020
P02766	TTHY_HUMAN	*TTR*	Transthyretin	3.89	0.0049
P22392	NDKB_HUMAN	*NME2*	Nucleoside diphosphate kinase B	3.70	0.0013
P13726	TF_HUMAN	*F3*	Tissue factor	3.70	0.0137
P10606	COX5B_HUMAN	*COX5B*	Cytochrome c oxidase subunit 5B, mitochondrial	3.20	0.0488
P04083	ANXA1_HUMAN	*ANXA1*	Annexin A1	3.04	0.0014
Q14019	COTL1_HUMAN	*COTL1*	Coactosin‐like protein	2.90	0.0070
P07355	ANXA2_HUMAN	*ANXA2*	Annexin A2	2.84	0.0012
P49840	GSK3A_HUMAN	*GSK3A*	Glycogen synthase kinase‐3 alpha	2.72	0.0439
Q15847	ADIRF_HUMAN	*ADIRF*	Adipogenesis regulatory factor	2.63	0.0045
P00403	COX2_HUMAN	*MT‐CO2*	Cytochrome c oxidase subunit 2	2.59	0.0006
P27105	STOM_HUMAN	*STOM*	Stomatin	2.41	0.0086
P15531	NDKA_HUMAN	*NME1*	Nucleoside diphosphate kinase A	2.41	0.0015
P40429	RL13A_HUMAN	*RPL13A*	60S ribosomal protein L13a	2.37	0.0311
Q9H444	CHM4B_HUMAN	*CHMP4B*	Charged multivesicular body protein 4b	2.37	0.0407
P35232	PHB_HUMAN	*PHB*	Prohibitin	2.37	0.0011
Q92688	AN32B_HUMAN	*ANP32B*	Acidic leucine‐rich nuclear phosphoprotein 32 family member B	2.36	0.0203
P31949	S10AB_HUMAN	*S100A11*	Protein S100‐A11	2.35	0.0192
Q13907	IDI1_HUMAN	*IDI1*	Isopentenyl‐diphosphate Delta‐isomerase 1	2.28	0.0390
P05387	RLA2_HUMAN	*RPLP2*	60S acidic ribosomal protein P2	2.27	0.0328
O76041	NEBL_HUMAN	*NEBL*	Nebulette	2.25	0.0219
O75131	CPNE3_HUMAN	*CPNE3*	Copine‐3	2.24	0.0182
O75828	CBR3_HUMAN	*CBR3*	Carbonyl reductase [NADPH] 3	2.22	0.0045
O00299	CLIC1_HUMAN	*CLIC1*	Chloride intracellular channel protein 1	2.19	0.0008
Q96HN2	SAHH3_HUMAN	*AHCYL2*	Adenosylhomocysteinase 3	2.18	0.0217
P53367	ARFP1_HUMAN	*ARFIP1*	Arfaptin‐1	2.16	0.0484
P45880	VDAC2_HUMAN	*VDAC2*	Voltage‐dependent anion‐selective channel protein 2	2.16	0.0205
P10644	KAP0_HUMAN	*PRKAR1A*	cAMP‐dependent protein kinase type I‐alpha regulatory subunit	2.14	0.0180
P0C0L5	CO4B_HUMAN	*C4B*	Complement C4‐B	2.10	0.0006
Q8NBX0	SCPDL_HUMAN	*SCCPDH*	Saccharopine dehydrogenase‐like oxidoreductase	2.08	0.0202
P01011	AACT_HUMAN	*SERPINA3*	Alpha‐1‐antichymotrypsin	2.07	0.0168
P26038	MOES_HUMAN	*MSN*	Moesin	2.07	0.0002
P15259	PGAM2_HUMAN	*PGAM2*	Phosphoglycerate mutase 2	2.04	0.0355
P10909	CLUS_HUMAN	*CLU*	Clusterin	2.03	0.0245
Q07020	RL18_HUMAN	*RPL18*	60S ribosomal protein L18	2.02	0.0081
P50995	ANX11_HUMAN	*ANXA11*	Annexin A11	2.02	0.0046
Q09666	AHNK_HUMAN	*AHNAK*	Neuroblast differentiation‐associated protein AHNAK	1.98	0.0012
P48681	NEST_HUMAN	*NES*	Nestin	1.98	0.0093
Q13938	CAYP1_HUMAN	*CAPS*	Calcyphosin	1.97	0.0084
P21796	VDAC1_HUMAN	*VDAC1*	Voltage‐dependent anion‐selective channel protein 1	1.97	0.0197
P04179	SODM_HUMAN	*SOD2*	Superoxide dismutase [Mn], mitochondrial	1.95	0.0008
P40121	CAPG_HUMAN	*CAPG*	Macrophage‐capping protein	1.94	0.0124
P62277	RS13_HUMAN	*RPS13*	40S ribosomal protein S13	1.94	0.0497
Q14254	FLOT2_HUMAN	*FLOT2*	Flotillin‐2	1.94	0.0164
Q09028	RBBP4_HUMAN	*RBBP4*	Histone‐binding protein RBBP4	1.93	0.0081
Q9ULC3	RAB23_HUMAN	*RAB23*	Ras‐related protein Rab‐23	1.92	0.0379
P13796	PLSL_HUMAN	*LCP1*	Plastin‐2	1.92	0.0130
P13073	COX41_HUMAN	*COX4I1*	Cytochrome c oxidase subunit 4 isoform 1, mitochondrial	1.90	0.0054
P30047	GFRP_HUMAN	*GCHFR*	GTP cyclohydrolase 1 feedback regulatory protein	1.90	0.0375
P15311	EZRI_HUMAN	*EZR*	Ezrin	1.89	0.0004
O15488	GLYG2_HUMAN	*GYG2*	Glycogenin‐2	1.86	0.0216
Q15417	CNN3_HUMAN	*CNN3*	Calponin‐3	1.84	0.0398
P61421	VA0D1_HUMAN	*ATP6V0D1*	V‐type proton ATPase subunit d 1	1.83	0.0356
Q01995	TAGL_HUMAN	*TAGLN*	Transgelin	1.82	0.0454
Q9Y3E1	HDGR3_HUMAN	*HDGFL3*	Hepatoma‐derived growth factor‐related protein 3	1.82	0.0157
Q96C23	GALM_HUMAN	*GALM*	Galactose mutarotase	1.82	0.0352
P50897	PPT1_HUMAN	*PPT1*	Palmitoyl‐protein thioesterase 1	1.80	0.0180
P08758	ANXA5_HUMAN	*ANXA5*	Annexin A5	1.79	0.0123
P25788	PSA3_HUMAN	*PSMA3*	Proteasome subunit alpha type‐3	1.77	0.0043
P08133	ANXA6_HUMAN	*ANXA6*	Annexin A6	1.76	0.0001
Q96DG6	CMBL_HUMAN	*CMBL*	Carboxymethylenebutenolidase homolog	1.76	0.0429
Q96G03	PGM2_HUMAN	*PGM2*	Phosphoglucomutase‐2	1.75	0.0067
Q9NPH2	INO1_HUMAN	*ISYNA1*	Inositol‐3‐phosphate synthase 1	1.75	0.0059
P04080	CYTB_HUMAN	*CSTB*	Cystatin‐B	1.75	0.0051
P62266	RS23_HUMAN	*RPS23*	40S ribosomal protein S23	1.75	0.0194
Q8TC26	TM163_HUMAN	*TMEM163*	Transmembrane protein 163	1.75	0.0261
P30041	PRDX6_HUMAN	*PRDX6*	Peroxiredoxin‐6	1.74	0.0018
Q3KQU3	MA7D1_HUMAN	*MAP7D1*	MAP7 domain‐containing protein 1	1.72	0.0421
Q8NBF2	NHLC2_HUMAN	*NHLRC2*	NHL repeat‐containing protein 2	1.72	0.0062
Q96AQ6	PBIP1_HUMAN	*PBXIP1*	Pre‐B‐cell leukemia transcription factor‐interacting protein 1	1.71	0.0152
Q9BPW8	NIPS1_HUMAN	*NIPSNAP1*	Protein NipSnap homolog 1	1.70	0.0383
P06865	HEXA_HUMAN	*HEXA*	Beta‐hexosaminidase subunit alpha	1.69	0.0181
Q7L9L4	MOB1B_HUMAN	*MOB1B*	MOB kinase activator 1B	1.69	0.0190
P84085	ARF5_HUMAN	*ARF5*	ADP‐ribosylation factor 5	1.67	0.0304
Q9BY32	ITPA_HUMAN	*ITPA*	Inosine triphosphate pyrophosphatase	1.67	0.0433
Q9H8H3	MET7A_HUMAN	*METTL7A*	Methyltransferase‐like protein 7A	1.66	0.0059
P29401	TKT_HUMAN	*TKT*	Transketolase	1.66	0.0030
O43399	TPD54_HUMAN	*TPD52L2*	Tumor protein D54	1.66	0.0496
P11766	ADHX_HUMAN	*ADH5*	Alcohol dehydrogenase class‐3	1.65	0.0246
O95336	6PGL_HUMAN	*PGLS*	6‐phosphogluconolactonase	1.65	0.0153
Q96Q06	PLIN4_HUMAN	*PLIN4*	Perilipin‐4	1.64	0.0455
Q9UL46	PSME2_HUMAN	*PSME2*	Proteasome activator complex subunit 2	1.64	0.0368
P51178	PLCD1_HUMAN	*PLCD1*	1‐phosphatidylinositol 4,5‐bisphosphate phosphodiesterase delta‐1	1.63	0.0057
P49721	PSB2_HUMAN	*PSMB2*	Proteasome subunit beta type‐2	1.63	0.0303
P55008	AIF1_HUMAN	*AIF1*	Allograft inflammatory factor 1	1.62	0.0234
P10768	ESTD_HUMAN	*ESD*	S‐formylglutathione hydrolase	1.62	0.0016
P20073	ANXA7_HUMAN	*ANXA7*	Annexin A7	1.62	0.0309
O75223	GGCT_HUMAN	*GGCT*	Gamma‐glutamylcyclotransferase	1.62	0.0402
Q00796	DHSO_HUMAN	*SORD*	Sorbitol dehydrogenase	1.62	0.0153
P49189	AL9A1_HUMAN	*ALDH9A1*	4‐trimethylaminobutyraldehyde dehydrogenase	1.62	0.0056
O14807	RASM_HUMAN	*MRAS*	Ras‐related protein M‐Ras	1.61	0.0455
P30626	SORCN_HUMAN	*SRI*	Sorcin	1.61	0.0493
Q9BQA1	MEP50_HUMAN	*WDR77*	Methylosome protein 50	1.61	0.0064
P63027	VAMP2_HUMAN	*VAMP2*	Vesicle‐associated membrane protein 2	1.60	0.0287
Q04760	LGUL_HUMAN	*GLO1*	Lactoylglutathione lyase	1.60	0.0040
Q96DB5	RMD1_HUMAN	*RMDN1*	Regulator of microtubule dynamics protein 1	1.60	0.0404
Q14118	DAG1_HUMAN	*DAG1*	Dystroglycan	1.59	0.0195
P61204	ARF3_HUMAN	*ARF3*	ADP‐ribosylation factor 3	1.58	0.0460
P43490	NAMPT_HUMAN	*NAMPT*	Nicotinamide phosphoribosyltransferase	1.58	0.0313
Q8N4P3	MESH1_HUMAN	*HDDC3*	Guanosine‐3′,5′‐bis(diphosphate) 3′‐pyrophosphohydrolase MESH1	1.57	0.0486
Q13683	ITA7_HUMAN	*ITGA7*	Integrin alpha‐7	1.57	0.0051
Q6IQ22	RAB12_HUMAN	*RAB12*	Ras‐related protein Rab‐12	1.55	0.0338
Q15599	NHRF2_HUMAN	*SLC9A3R2*	Na(+)/H(+) exchange regulatory cofactor NHE‐RF2	1.52	0.0300
P09211	GSTP1_HUMAN	*GSTP1*	Glutathione S‐transferase P	1.50	0.0136
P25786	PSA1_HUMAN	*PSMA1*	Proteasome subunit alpha type‐1	1.50	0.0402
P27816	MAP4_HUMAN	*MAP4*	Microtubule‐associated protein 4	1.50	0.0219

Downregulated proteins					
P23468	PTPRD_HUMAN	*PTPRD*	Receptor‐type tyrosine‐protein phosphatase delta	0.67	0.0323
Q7KZF4	SND1_HUMAN	*SND1*	Staphylococcal nuclease domain‐containing protein 1	0.67	0.0434
O75122	CLAP2_HUMAN	*CLASP2*	CLIP‐associating protein 2	0.67	0.0368
Q9H0E2	TOLIP_HUMAN	*TOLLIP*	Toll‐interacting protein	0.66	0.0124
Q15111	PLCL1_HUMAN	*PLCL1*	Inactive phospholipase C‐like protein 1	0.66	0.0275
P36551	HEM6_HUMAN	*CPOX*	Oxygen‐dependent coproporphyrinogen‐III oxidase, mitochondrial	0.66	0.0115
Q08380	LG3BP_HUMAN	*LGALS3BP*	Galectin‐3‐binding protein	0.65	0.0277
P50453	SPB9_HUMAN	*SERPINB9*	Serpin B9	0.65	0.0340
O95670	VATG2_HUMAN	*ATP6V1G2*	V‐type proton ATPase subunit G 2	0.65	0.0475
O43615	TIM44_HUMAN	*TIMM44*	Mitochondrial import inner membrane translocase subunit TIM44	0.65	0.0490
Q96RU3	FNBP1_HUMAN	*FNBP1*	Formin‐binding protein 1	0.65	0.0382
P14866	HNRPL_HUMAN	*HNRNPL*	Heterogeneous nuclear ribonucleoprotein L	0.64	0.0460
Q86VS8	HOOK3_HUMAN	*HOOK3*	Protein Hook homolog 3	0.63	0.0045
Q9NP81	SYSM_HUMAN	*SARS2*	Serine—tRNA ligase, mitochondrial	0.63	0.0464
Q01433	AMPD2_HUMAN	*AMPD2*	AMP deaminase 2	0.62	0.0035
O95757	HS74L_HUMAN	*HSPA4L*	Heat shock 70 kDa protein 4 L	0.62	0.0322
Q9GZM8	NDEL1_HUMAN	*NDEL1*	Nuclear distribution protein nudE‐like 1	0.62	0.0150
Q96B97	SH3K1_HUMAN	*SH3KBP1*	SH3 domain‐containing kinase‐binding protein 1	0.62	0.0109
Q04323	UBXN1_HUMAN	*UBXN1*	UBX domain‐containing protein 1	0.62	0.0083
Q9H9P8	L2HDH_HUMAN	*L2HGDH*	L‐2‐hydroxyglutarate dehydrogenase, mitochondrial	0.61	0.0360
Q5T4S7	UBR4_HUMAN	*UBR4*	E3 ubiquitin‐protein ligase UBR4	0.60	0.0310
Q92609	TBCD5_HUMAN	*TBC1D5*	TBC1 domain family member 5	0.60	0.0143
P46379	BAG6_HUMAN	*BAG6*	Large proline‐rich protein BAG6	0.59	0.0202
Q04609	FOLH1_HUMAN	*FOLH1*	Glutamate carboxypeptidase 2	0.59	0.0275
P48147	PPCE_HUMAN	*PREP*	Prolyl endopeptidase	0.59	0.0143
P02787	TRFE_HUMAN	*TF*	Serotransferrin	0.59	0.0027
Q8NBJ7	SUMF2_HUMAN	*SUMF2*	Inactive C‐alpha‐formylglycine‐generating enzyme 2	0.58	0.0450
Q9BXJ9	NAA15_HUMAN	*NAA15*	N‐alpha‐acetyltransferase 15, NatA auxiliary subunit	0.58	0.0233
Q13617	CUL2_HUMAN	*CUL2*	Cullin‐2	0.58	0.0488
Q8N7J2	AMER2_HUMAN	*AMER2*	APC membrane recruitment protein 2	0.57	0.0148
Q96FC7	PHIPL_HUMAN	*PHYHIPL*	Phytanoyl‐CoA hydroxylase‐interacting protein‐like	0.56	0.0067
Q15438	CYH1_HUMAN	*CYTH1*	Cytohesin‐1	0.56	0.0019
Q8IXJ6	SIR2_HUMAN	*SIRT2*	NAD‐dependent protein deacetylase sirtuin‐2	0.56	0.0062
Q9C0D3	ZY11B_HUMAN	*ZYG11B*	Protein zyg‐11 homolog B	0.56	0.0181
P48426	PI42A_HUMAN	*PIP4K2A*	Phosphatidylinositol 5‐phosphate 4‐kinase type‐2 alpha	0.55	0.0008
Q6L8Q7	PDE12_HUMAN	*PDE12*	2′,5′‐phosphodiesterase 12	0.55	0.0242
Q9C0E8	LNP_HUMAN	*LNPK*	Endoplasmic reticulum junction formation protein lunapark	0.54	0.0241
Q13619	CUL4A_HUMAN	*CUL4A*	Cullin‐4A	0.53	0.0284
Q3ZCW2	LEGL_HUMAN	*LGALSL*	Galectin‐related protein	0.53	0.0445
P60228	EIF3E_HUMAN	*EIF3E*	Eukaryotic translation initiation factor 3 subunit E	0.53	0.0091
Q9Y276	BCS1_HUMAN	*BCS1L*	Mitochondrial chaperone BCS1	0.53	0.0427
Q8N111	CEND_HUMAN	*CEND1*	Cell cycle exit and neuronal differentiation protein 1	0.52	0.0320
O00505	IMA4_HUMAN	*KPNA3*	Importin subunit alpha‐4	0.52	0.0167
Q9NTM9	CUTC_HUMAN	*CUTC*	Copper homeostasis protein cutC homolog	0.52	0.0427
Q16773	KAT1_HUMAN	*KYAT1*	Kynurenine‐oxoglutarate transaminase 1	0.52	0.0048
O76094	SRP72_HUMAN	*SRP72*	Signal recognition particle subunit SRP72	0.52	0.0355
Q9H9Q2	CSN7B_HUMAN	*COPS7B*	COP9 signalosome complex subunit 7b	0.52	0.0287
O95292	VAPB_HUMAN	*VAPB*	Vesicle‐associated membrane protein‐associated protein B/C	0.52	0.0074
Q5TCQ9	MAGI3_HUMAN	*MAGI3*	Membrane‐associated guanylate kinase, WW and PDZ domain‐containing protein 3	0.51	0.0224
Q9NR45	SIAS_HUMAN	*NANS*	Sialic acid synthase	0.51	0.0040
Q9Y2J0	RP3A_HUMAN	*RPH3A*	Rabphilin‐3A	0.51	0.0192
Q7Z4S6	KI21A_HUMAN	*KIF21A*	Kinesin‐like protein KIF21A	0.51	0.0033
P19823	ITIH2_HUMAN	*ITIH2*	Inter‐alpha‐trypsin inhibitor heavy chain H2	0.50	0.0448
P28676	GRAN_HUMAN	*GCA*	Grancalcin	0.50	0.0177
Q9UIA9	XPO7_HUMAN	*XPO7*	Exportin‐7	0.50	0.0093
Q9UPV7	PHF24_HUMAN	*PHF24*	PHD finger protein 24	0.49	0.0332
Q9NQW6	ANLN_HUMAN	*ANLN*	Anillin	0.48	0.0098
O60262	GBG7_HUMAN	*GNG7*	Guanine nucleotide‐binding protein G(I)/G(S)/G(O) subunit gamma‐7	0.48	0.0169
O75689	ADAP1_HUMAN	*ADAP1*	Arf‐GAP with dual PH domain‐containing protein 1	0.48	0.0085
O75208	COQ9_HUMAN	*COQ9*	Ubiquinone biosynthesis protein COQ9, mitochondrial	0.45	0.0050
A5YM72	CRNS1_HUMAN	*CARNS1*	Carnosine synthase 1	0.44	0.0063
Q9UDY2	ZO2_HUMAN	*TJP2*	Tight junction protein ZO‐2	0.43	0.0382
Q96GW9	SYMM_HUMAN	*MARS2*	Methionine—tRNA ligase, mitochondrial	0.42	0.0091
P20916	MAG_HUMAN	*MAG*	Myelin‐associated glycoprotein	0.42	0.0025
Q96FJ2	DYL2_HUMAN	*DYNLL2*	Dynein light chain 2, cytoplasmic	0.41	0.0078
O94967	WDR47_HUMAN	*WDR47*	WD repeat‐containing protein 47	0.40	0.0130
Q8TAM6	ERMIN_HUMAN	*ERMN*	Ermin	0.39	0.0217
P02689	MYP2_HUMAN	*PMP2*	Myelin P2 protein	0.28	0.0170
Q96HU8	DIRA2_HUMAN	*DIRAS2*	GTP‐binding protein Di‐Ras2	0.26	0.0396
P53597	SUCA_HUMAN	*SUCLG1*	Succinate—CoA ligase [ADP/GDP‐forming] subunit alpha, mitochondrial	0.23	0.0307

*Note*: FC < 1.5, *p* value <0.05, total identified proteins available via ProteomeXchange with identifier PXD038322.

In order to relate the DEPs to the specific neuronal, microglial and/or astroglial cell populations, we crossed them with lists of proteins preferentially expressed in each cell type, as well as with lists of proteins which interacts with Aβ (APP interactome) and Tau (MAPT interactome) to see their involvement in the pathology (for details, see Online Resource 11). Thus, cell cycle exit and neuronal differentiation protein 1 (CEND1), WDR47, and DIRAS2 were identified as DEPs and preferentially expressed in neurons. Nineteen proteins were recognized as DEPs and preferentially expressed in microglia. Specifically, Annexin A5 (ANXA5) was associated with both pathological markers and microglia, and proteasome activator complex subunit 2 (PSME2) and galectin‐3‐binding protein (LGALS3BP) were associated with Aβ and microglia. Eighteen proteins were linked to astrocytes. The marker clusterin (CLU) was related to both pathological markers, Flotillin‐2 (FLOT2) to Tau interactions and astrocytes, and peroxiredoxin‐6 (PRDX6) to Aβ and astrocytes (Table 3; Online Resource 12).

**TABLE 3:** Identified proteins from DEPs that interact with pathological proteins and expressed in neurons, microglia, and astrocytes.

DEPs‐neurons	DEPs‐neurons‐Aβ	DEPs‐neurons‐tau	DEPs‐neurons‐Aβ‐tau
CEND1, WDR47, DIRAS2	‐	‐	‐

DEPs‐microglia	DEPs‐Microglia‐Aβ	DEPs‐Microglia‐Tau	DEPs‐Microglia‐Aβ‐Tau
PLD3, ANXA1, COTL1, S100A11, CLIC1, SERPINA3, MSN, ANXA11, CAPG, LCP1, GALM, PPT1, ISYNA1, CSTB, HEXA, AIF1, SORD, SH3KBP1	PSME2, LGALS3BP	‐	ANXA5

DEPs‐astrocytes	DEPs‐Astrocytes‐Aβ	DEPs‐Astrocytes‐Tau	DEPs‐Astrocytes‐Aβ‐Tau
F3, NEBL, CBR3, AHCYL2, NES, CNN3, AGLN, CMBL, PBXIP1, MRAS, DAG1, ITGA7, MAP4, FOLH1, AMER2, PHYHIPL, LGALSL, TJP2	FLOT2	PRDX6	CLU

*Note*: Four main groups are presented: proteins preferentially expressed in cell type, proteins preferentially expressed in cell type that interact with Aβ, proteins preferentially expressed in cell type that interact with tau, and proteins preferentially expressed that interact with both markers are shown.

SynGo analysis revealed certain synaptic alterations in AD (29 proteins of 178 DEPs) with a clear effect on the synaptic vesicle system (Table 4; for detailed analysis, see Online Resource 10). On the other hand, Metascape analysis revealed affected processes such as cellular responses to stress, regulation of proteolysis, regulation of vesicle‐mediated transport, apoptotic signaling pathway or response to wounding, among others (Table 5; for detailed analysis, see Online Resources 11 and 12).

**TABLE 4:** SynGo analysis revealed synaptic affectation in AD.

GO term ID	GO domain	GO term name	FDR corrected *p* value	Genes
GO:0045202	CC	Synapse	0.000103167	*FLOT2; PRKAR1A; MAGI3; RPLP2; RPS13; RPL18; RPS23; CLU; RPL13A; HNRNPL; PHB; ANXA1; CADPS; VDAC1; ANXA5; RPH3A; ATP6V1G2; VAMP2; TMEM163; ATP6V0D1; CNTN1; CYTH1; PTPRD; DYNLL2; CNN3; DAG1; EIF3E*
GO:0098793	CC	Presynapse	0.000139198	*CADPS; VDAC1; FLOT2; PHB; ANXA5; RPH3A; ATP6V1G2; VAMP2; TMEM163; ATP6V0D1; CNTN1; CYTH1; PTPRD; RPL13A; RPL18; RPLP2; RPS13*
GO:0048787	CC	Presynaptic active zone membrane	0.027329597	*VDAC1; FLOT2; PHB*
GO:0030672	CC	Synaptic vesicle membrane	0.002092777	*ANXA5; RPH3A; ATP6V1G2; VAMP2; TMEM163; ATP6V0D1*
GO:0030285	CC	Integral component of synaptic vesicle membrane	0.027329597	*VAMP2; TMEM163; ATP6V0D1*
GO:0098794	CC	Postsynapse	0.027329597	*DYNLL2; CNN3; DAG1; PHB; RPS13; EIF3E; VDAC1; CNTN1; RPL13A; RPL18; RPLP2; RPS23*
GO:0099504	BP	Synaptic vesicle cycle	0.027829211	*VAMP2; CADPS; RPH3A; TMEM163; ATP6V0D1; ATP6V1G2*
GO:0140236	BP	Translation at presynapse	0.01123458	*RPL13A; RPL18; RPLP2; RPS13*
GO:0140242	BP	Translation at postsynapse	0.01123458	*RPL13A; RPL18; RPLP2; RPS13*

*Note*: Outstanding information about SynGo analysis including the GO information, false discovery rate and genes involved.

Abbreviations: BP, biological process; CC, cellular component; FDR, false discovery rate; GO, GeneOntology.

**TABLE 5:** Functional analysis by Metascape

Term	Description	Log (P)	Log(q‐value)	Proteins
R‐HSA‐2262752	Cellular responses to stress	−9.55086	−5.355	ATP6V1G2, COX4I1, COX5B, GSK3A, GSTP1, COX2, PSMA1, PSMA3, PSMB2, PSME2, RBBP4, RPL18, RPLP2, RPS13, RPS23, SOD2, TKT, CUL2, ATP6V0D1, PRDX6, HSPA4L, RPL13A, DYNLL2
GO:0030162	Regulation of proteolysis	−5.65205	−2.406	SERPINA3, ANXA2, C4B, CD44, CLU, CSTB, F3, GSK3A, ITIH2, SERPINB9, PSMA3, PSME2, BAG6, ANP32B, SIRT2, UBXN1, ZYG11B
GO:0032386	Regulation of intracellular transport	−5.31044	−2.263	ANXA2, STOM, GSK3A, LCP1, MSN, VAMP2, EZR, ANP32B, ARFIP1, RAB23, NDEL1, DAG1, SRI
GO:0048260	Positive regulation of receptor‐mediatedendocytosis	−4.81393	−1.928	ANXA2, CLU, PPT1, TF, TBC1D5, GLO1
GO:0060627	Regulation of vesicle‐mediated transport	−4.72155	−1.926	ANXA1, ANXA2, C4B, CLU, MSN, PPT1, VAMP2, TF, EZR, TBC1D5, CLASP2, ARFIP1, RAB12, BCS1L, FLOT2, SRP72, BAG6, SLC9A3R2, CHMP4B
GO:0030036	Actin cytoskeleton organization	−4.69625	−1.926	AIF1, ANXA1, CAPG, CNN3, LCP1, PRKAR1A, TF, EZR, NEBL, MRAS, SH3KBP1, ANLN, ERMN, CLASP2, HDGFL3, NDEL1, HOOK3
GO:0097190	Apoptotic signaling pathway	−4.46144	−1.794	ANXA6, CLU, GSK3A, SOD2, VDAC2, BAG6, CUL4A, CUL2, GGCT, AIF1, CD44, GSTP1, ICAM1, VDAC1, EZR, UBXN1, NDEL1, MAGI3, BCS1L, PHB1, TIMM44, DAG1
GO:0006914	Autophagy	−4.31508	−1.718	ANXA7, CLU, PIP4K2A, TBC1D5, SIRT2, RAB23, TOLLIP, CHMP4B, RAB12, ATP6V0D1, HOOK3
GO:0009611	Response to wounding	−4.2287	−1.678	AIF1, ANXA5, ANXA6, CD44, CLIC1, DAG1, F3, MAG, SOD2, LNPK, CHMP4B

*Note*: Proteins related to main affected pathways and biological processes.

**FIGURE 4:**
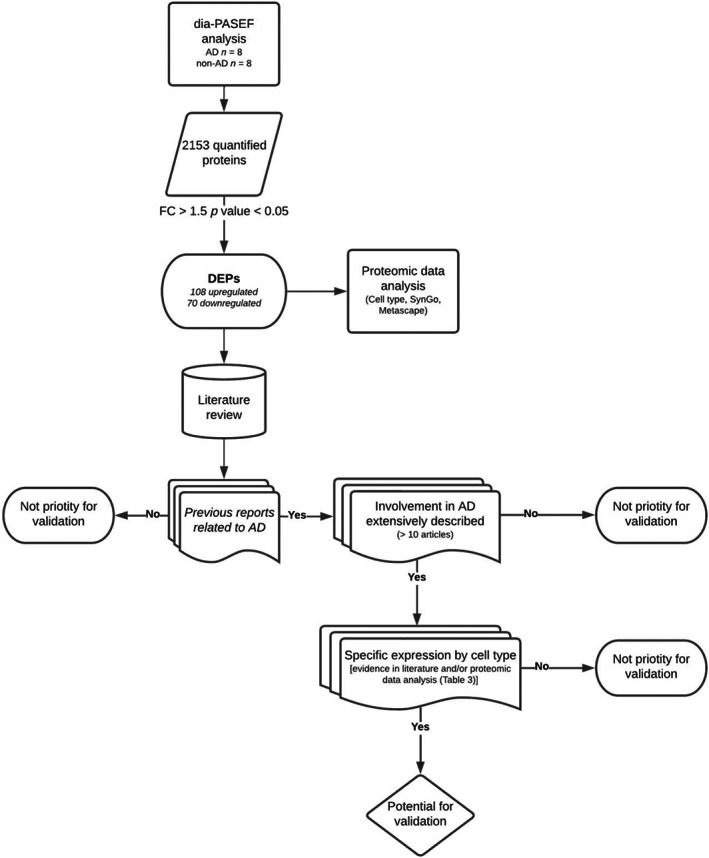
Procedure for proteomic data analysis and criteria for protein selection validation. In a first step, dia‐PASEF analysis of human AC samples revealed 2153 proteins. After applying restricted condition of FC > 1.5 and *p* value <0.05, 178 proteins were identified as DEPs and cell type expression, SynGo and Metascape analyses were performed (data shown in Tables 3, 4, 5). Then, literature review of DEPs was carried out in order to select proteins for validation. Proteins were chosen based on three main criteria: previous evidence linking protein and AD must be reported; proteins widely described in the disease were excluded; and potential relation or expression in the studied cell types (neurons, microglia, and astrocytes) was also considered.

The selection of proteins for validation was based on available literature and FC threshold. Proteins with no evidence or relation with the disease were excluded. Since the aim of the study was to provide new insights about AD in AC, well‐known proteins associated with the pathology were also excluded. Furthermore, potential relation or expression in the studied cell types (neurons, microglia, and astrocytes) was also considered for protein selection (Figure 4). Considering these criteria, Arf‐GAP with dual PH domain‐containing protein 1 (ADAP1), CEND1, and ANXA2 were selected for neuronal; chloride intracellular channel protein 1 (CLIC1) and ANXA5 for microglial; and Annexin A1 (ANXA1) and PRDX6 for astroglial evaluation by confocal analysis.

### 3.5 Neuronal and glial responses to pathology in the AC

**FIGURE 5:**
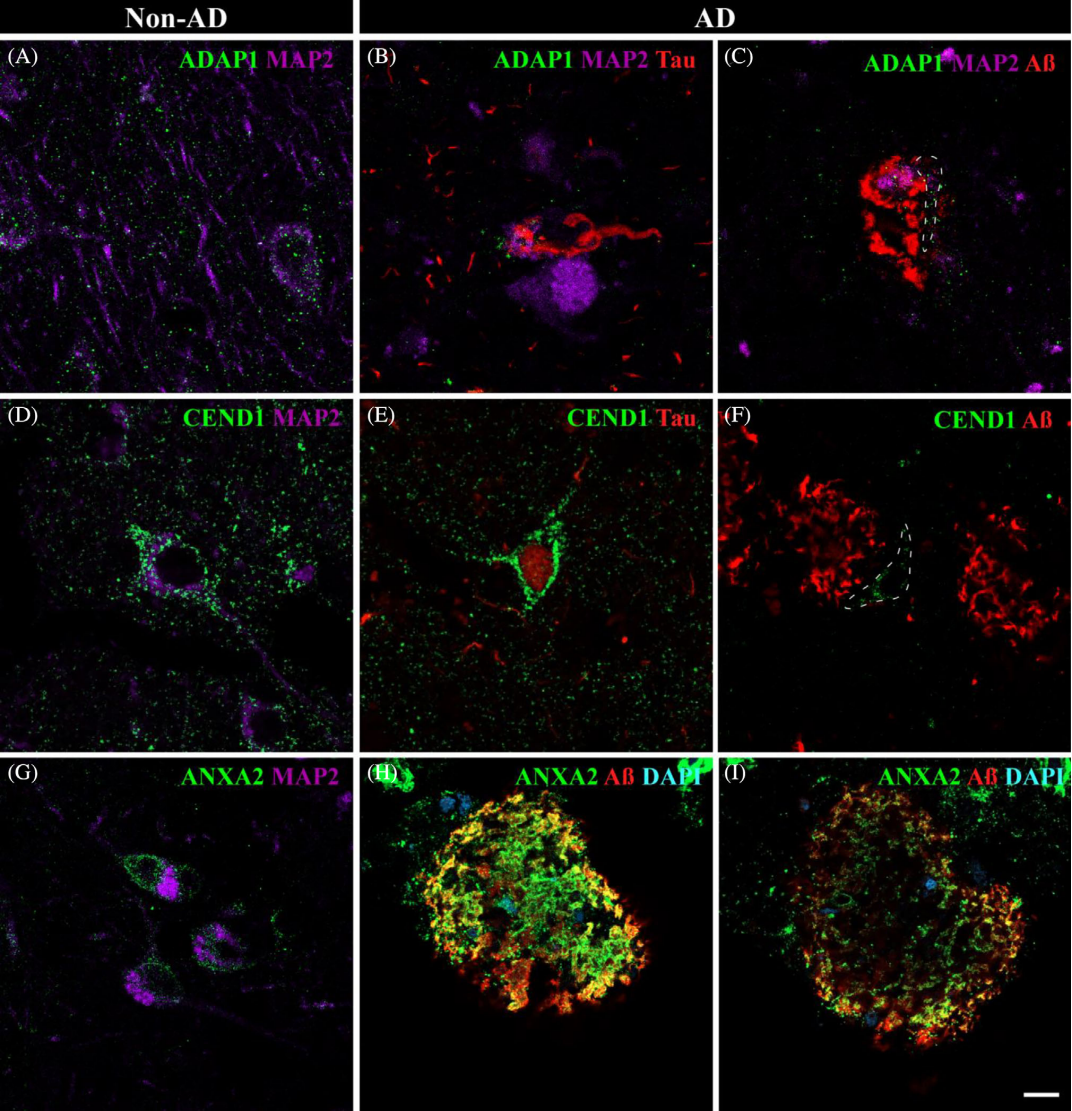
Neuronal involvement in the amygdaloid complex nuclei in AD: ADAP1, CEND1, and ANXA2. Triple immunofluorescence against ADAP1 (A–C), CEND1 (D,E), ANXA2 (G–I), and pathological markers. In non‐AD, ADAP1 (A, green) was mainly associated with vesicles in axons and dendrites, although it was also observed in soma. CEND1 (D, green) revealed neuronal expression in non‐AD samples. ADAP1 expression was drastically reduced in AD (B,C), with spatial coexpression with Tau (red) and MAP2 (purple) in the soma (B). Neurons close to Aβ (C, dashed line) presented a reduced number of ADAP1 vesicles in the soma and axon. A reduced number of CEND1‐stained neurons was observed in AD (E,F). CEND1 staining was remarkably associated with Tau deposits (E) compared with neurons near Aβ plaques (F, dashed line). ANXA2 (G, green) expression in neurons was identified in non‐AD samples. In AD, ANXA2 expression was increased close to Aβ (red) deposits (H,I). ANXA2 staining was higher on the outside of the plaques (H) than on the inside (I). Scale bar = 10 μm.

According to the proteomic analysis, ADAP1 and CEND1 were identified as downregulated, while ANXA2 was identified as upregulated by dia‐PASEF analysis. ADAP1 expression was identified not only in the soma but also associated with dendrites and axons in non‐AD samples (Figure 5A). However, ADAP1 labeling was dramatically reduced in AD samples (Figure 5B,C). Its expression was observed to be associated with Tau (Figure 5B) and soma (Figure 5C, dashed line). Likewise, CEND1 was widely expressed in neurons in non‐AD samples (Figure 5D). Nevertheless, few neurons were labeled with CEND1 in AD samples (Figure 5E,F). Interestingly, when labeling was identified in neurons in the vicinity of Aβ, CEND1 expression was reduced (Figure 5F, dashed line) compared to that surrounding Tau deposits (Figure 5E). ANXA2 was expressed by neurons in non‐AD samples (Figure 5G), and qualitatively, the ANXA2 intensity of labeling was higher in AD samples (Figure 5H,I). ANXA2 was closely distributed with Aβ plaques (Figure 5H,I), being more intense in the periphery of the plaques (Figure 5H) than inside (Figure 5I).

**FIGURE 6:**
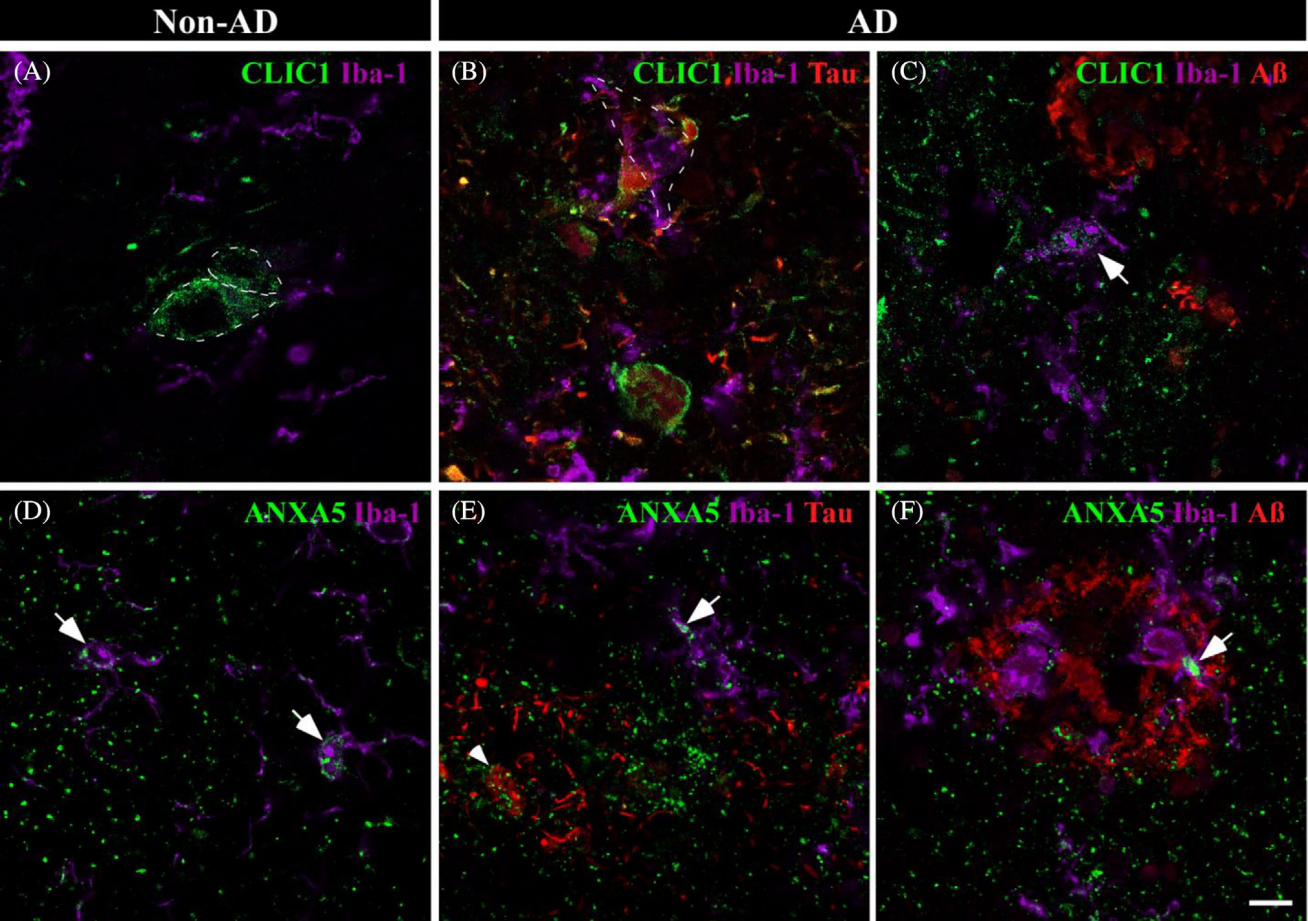
Microglial involvement in amygdaloid pathology in AD. Immunofluorescences against CLIC1 (A–C) and ANXA5 (D–F) and pathological markers are shown. In non‐AD samples, CLIC1 (A, green) labeling suggested possible expression in neurons (dashed line). In AD, CLIC1 colocalized intimately with Tau pathology (B, red) and microglia (purple, dashed line). Additionally, CLIC1 expression was observed in the microglial cells nearest to Aβ plaques (red) (C, arrow). ANXA5 (D, green) was related to microglia (purple, arrow) in non‐AD tissue. Microglial ANXA5 expression was increased in AD (E,F, arrow) with a closed spatial expression with Aβ plaques (E, reed) and Tau deposits (F, arrowhead). Scale bar = 10 μm.

CLIC1 and ANXA5 were assessed as upregulated by proteomic analysis. In non‐AD samples, CLIC1 labeling suggested possible expression in neurons (Figure 6A, dashed line). In AD, we observed two different situations: first, CLIC1 colocalized with Tau deposits, with microglia frequently present close to those affected neurons (Figure 6B, dashed line), and second, microglia expressed CLIC1 in the vicinity of Aβ (Figure 6C, arrow). On the other hand, ANXA5 was expressed in microglia in non‐AD samples (Figure 6D, arrow) and more intensely expressed in AD samples (Figure 6E,F, arrow). ANXA5 was frequently observed with Tau deposits (Figure 6E, arrowhead), whereas ANXA5‐microglia coexpression was closely associated with Aβ in AD samples (Figure 6F, arrow).

**FIGURE 7:**
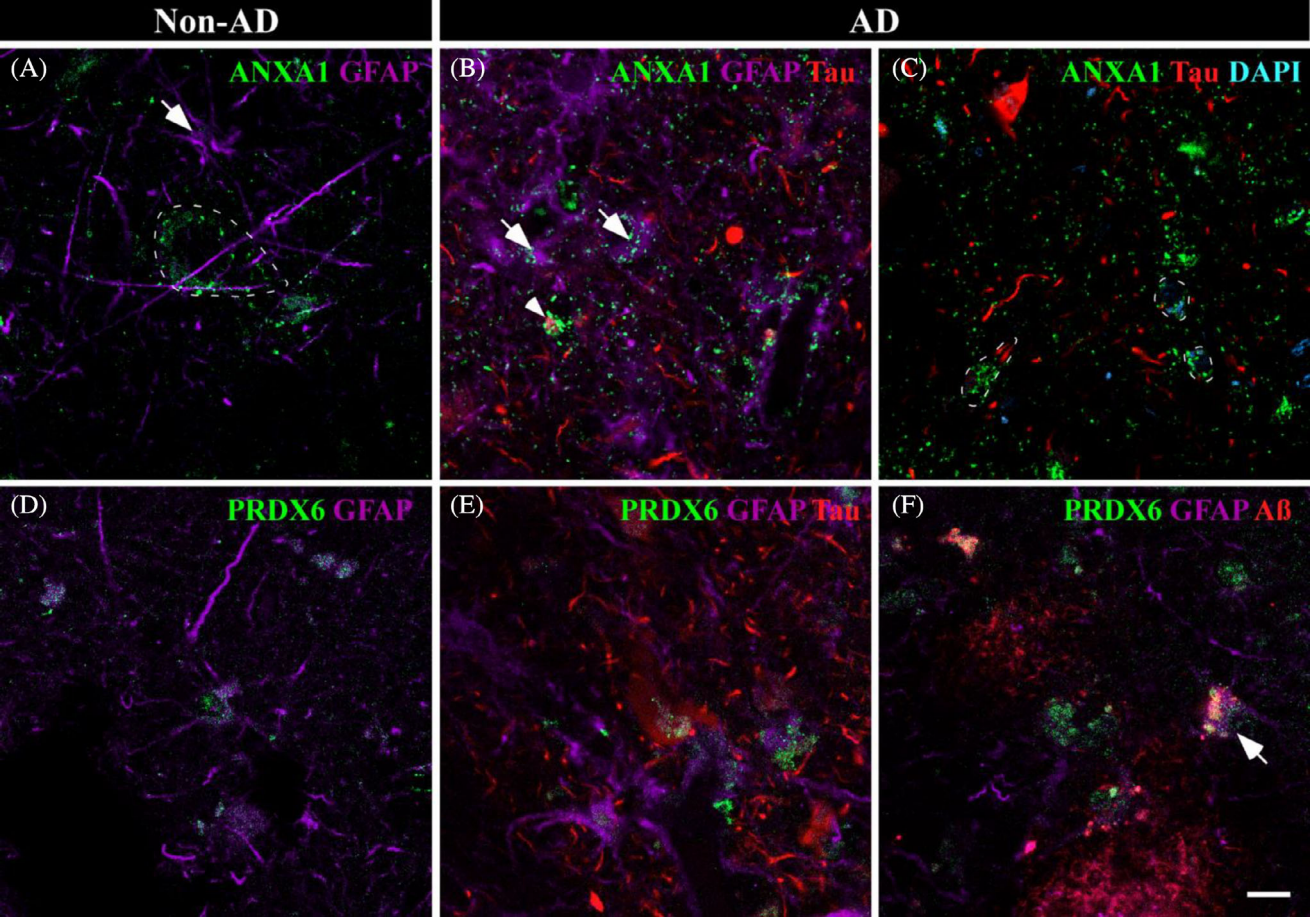
Astroglial participation in AD. Immunofluorescences against ANXA1 and PRDX6 are shown in non‐AD (A and D, respectively) and AD (B,C and E,F, respectively) samples. In non‐AD samples, ANXA1 (A, green) was expressed in neurons (dashed line) and in astrocytes to a lesser extent (purple, arrow). In AD, ANXA1 expression in astrocytes was increased (B, arrow), and ANXA1 was coexpressed with Tau deposits (B, arrowhead). Frequently, neurons with slight Tau staining expressed increased levels of ANXA1 (C, dashed line). PRDX6 (green) expression by astrocytes (purple) was observed in non‐AD (D) and AD (E,F) samples. PRDX6 was related to Tau (red) (E) and Aβ (red) (F) pathology. Scale bar = 10 μm.

Concerning ANXA1 and PRDX6, dia‐PASEF analysis revealed upregulated expression in AD samples. In non‐AD samples, ANXA1 was expressed in neurons (Figure 7A, dashed line) and, to a lesser extent, in astrocytes (Figure 7A, arrow). Increased ANXA1 was observed in astrocytes in AD samples (Figure 7B, arrow) with tight spatial coexpression with Tau (Figure 7B, arrowhead). Frequently, neurons with Tau deposits were marked with ANXA1 (Figure 7C, dashed line). On the other hand, PRDX6 was associated with astrocytes in non‐AD and AD samples (Figure 7D–F, respectively). Colocalization with pathological markers was observed (Figure 7E,F), with remarkable coexpression with small accumulations of Aβ (Figure 7F, arrow).

## 4 DISCUSSION

The present work includes a dual approach using stereological and proteomic techniques with the aim of assessing neuronal and glial involvement in the AC in AD. Synaptic alterations as well as the potential participation of glial cells in response to pathology have been identified as particularly relevant in AC pathology in AD.

Amygdala volume reduction has been postulated as a diagnostic criterion in AD [19], since amygdala atrophy has been described as comparable to that in the hippocampus [13]. Specifically, histological analysis and diffeomorphometry highlight the BL and BM as the most affected nuclei in AD [20, 37], and it is also linked to neuronal loss in the different nuclei analyzed [21, 22, 38]. In the present study, amygdala atrophy was confirmed, and the Co and La were identified as the most affected nuclei (Figure 1C). However, the volume reduction was not associated with differences in neuronal populations (Figure 2C) but with neuropil, which could be related to synaptic alterations, as highlighted by proteomic data analysis (Table 4). This is in consonance with the reduction in intrinsic connections in the BLA described in the literature [39]. The discrepancy with previous studies could be explained since no specific cell type markers have been employed to identify neurons, establishing a possible bias in the analysis.

**FIGURE 8:**
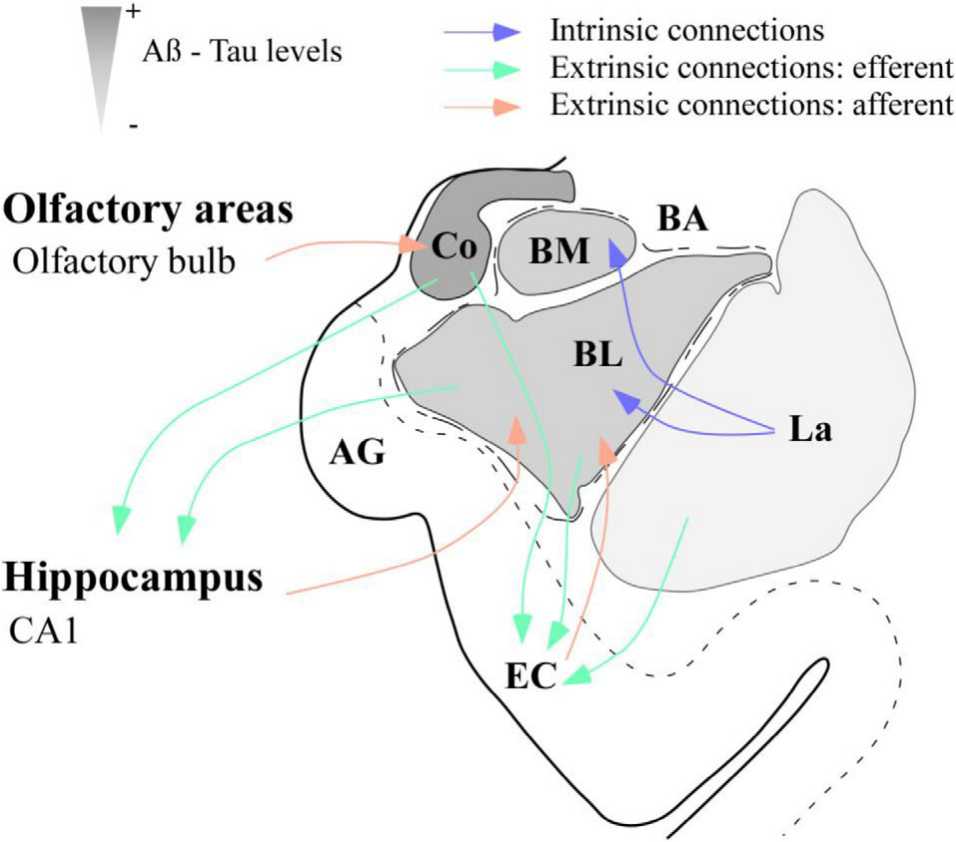
Amygdaloid complex as a “switch” in AD. Scheme of the amygdaloid complex (AC) and its main connections with olfactory areas, the hippocampus, and the entorhinal cortex (EC). Different amygdaloid nuclei are represented in grayscale from more (darker) to less (weaker) affected by pathology. Efferences and afferences regarding olfactory areas, CA1 and the EC might act as vehicles for pathology from and to the AC. AG, ambiens gyrus; BL, basolateral nucleus; BM, basomedial nucleus; Co, cortical nucleus; La, lateral nucleus.

In addition, the glial population has been described to be affected in AD. A reduction in glial cells has been identified in the BL and Co [21], and morphological changes have been described in the latest stages of AD [40]. However, the analysis was conducted with cresyl violet, and glia were differentiated from neurons by morphology, without distinguishing between astrocytes and microglia. Here, we conducted separate analyses of microglia and astrocytes with specific markers, resulting in an increase in astrocytes (Figure 2I) and no variation in the microglial population (Figure 2F). The increase in the number of astrocytes, as well as the microgliosis observed in all analyzed nuclei, might be generated as a response to pathology. Pathological markers have been described to affect different nuclei, since plaques are predominantly present in the BLA, whereas tangles are mainly present in the corticomedial complex [41, 42, 43]. However, we observed a similar distribution pattern of pathology in the AC concerning Tau and Aβ, which appeared as a gradient from the cortical to lateral areas, with the Co, BM, and BL being more affected than the La (Figure 3C,D). Furthermore, TDP‐43‐P pathology observed in the amygdala nuclei resembles Tau and Aβ distribution (Online Resource 10). The involvement of these nuclei could be related to the spread of the disease via connections with the hippocampus and/or olfactory areas [44]. Pathology might propagate from the olfactory and hippocampal areas (early affected in AD) to the Co and BL, respectively. The projections from the Co to CA1 and layer I‐II of the entorhinal cortex (EC), together with the loops established between CA1‐BA‐CA1 and layer V‐BL‐layer III to V of the EC (diffuse projections) [16, 44, 45], might indicate that the AC is a regulator of pathology distribution in these areas [46] (Figure 8).

Considering proteomic data analysis, neuronal and glial implications in amygdala pathology were evaluated by confocal microscopy. In this sense, ADAP1, CEND1, and ANXA2 revealed a close linkage with neurons, whereas ANXA1, ANXA5, CLIC1, and PRDX6 may have a potential role in the pathology response through glia.

ADAP1 is a brain‐specific GTPase‐activating protein and a member of the ADP ribosylation factor family; ADAP1 is localized in axonal processes and is frequently associated with presynaptic vesicles. ADAP1 participates in dendritic differentiation since its downregulation inhibits dendritic branching and reduces the length of dendrites, with no effect on axon morphology [47]. Recently, a pathological role of ADAP1 has been described because the increase in its expression has been identified as a response to Aβ, resulting in synaptic dysfunction and negative regulation of memory formation in mouse models [48, 49]. However, to the best of our knowledge, only one previous report has confirmed the increased expression of ADAP1 in human tissue by immunostaining [50]. In contrast, our results revealed a reduction in ADAP1 expression (FC = 0.47574, *p* value = 0,009) in human amygdala AD samples identified by dia‐PASEF. Furthermore, immunofluorescence revealed reduced labeling in AD samples (Figure 5B,C), possibly because of synaptic dysfunction. These results highlight the need for further studies to elucidate the involvement of ADAP1 in human AD.

CEND1 is a brain‐specific protein that plays an important role in neuronal differentiation [51]. Previous data have reported that CEND1 expression is decreased in the brains of AD mice, resulting in synaptic dysfunction [52]. Here, we found that CEND1 is decreased in AD human samples by dia‐PASEF analysis, confirming previous results in animal models. Although neurons labeled by CEND1 were scarce in AD samples, reduced expression was notable in neurons near Aβ plaques (Figure 5F) compared to Tau deposits (Figure 5E), suggesting a potential involvement of Aβ in CEND1 expression. The reduction in CEND1 in AD may potentiate synaptic dysfunction in human amygdala pathology.

ANXA2 has been described to participate in the redistribution of Tau under pathological conditions [53] and to facilitate autophagosome‐lysosome fusion to reduce Aβ accumulation [54]. Here, we observed increased expression of ANXA2 in AD human amygdala samples according to proteomic data, which is consistent with previous results from our laboratory [28]. ANXA2 was associated with neurons in non‐AD samples (Figure 5G) and particularly with Aβ in AD samples (Figure 6H,I), suggesting the possible engulfment of this marker in the autophagosome‐lysosome system.

CLIC1 is an intracellular chloride channel proposed as a potential marker of neurodegenerative processes [55]. It has been described to participate in the microglial activation induced by Aβ, causing a harmful phenotype that produces reactive oxygen species and, consequently, neuronal death [56]. The blockage of CLIC1 promotes Aβ phagocytosis, inhibiting the neurotoxic phenotype of microglia [57]. In this sense, the increase in CLIC1 observed by proteomic analysis might be linked to inflammatory and neurotoxic processes. CLIC1 expression observed in microglia in AD samples (Figure 6C, arrow) and microglia disposed in close contact with tangles (Figure 6B, dashed line) might induce apoptosis.

A protective role of ANXA5 against Ca^2+^‐induced damage and reducing Aβ toxicity has been highlighted [58]. Furthermore, ANXA5 has been evaluated as a potential biomarker for AD since its plasma levels are increased in AD [59] and as a potential candidate for monitoring the progression of the disease [60]. A previous report in our laboratory revealed enriched ANXA5 in AD extracts, which was especially noticeable surrounding Aβ plaques [28]. Our results confirmed the elevated expression of ANXA5 in AD samples and identified ANXA5 expression in microglia (Figure 6D, arrow). This increased expression and the ANXA5 interaction with pathological markers (Figure 6E,F) observed by immunofluorescence suggest an attempt to reduce Tau and Aβ toxicity by microglia.

ANXA1 is a proresolving protein that modulates microglial activation and stimulates the phagocytosis of apoptotic neurons by microglia by acting as an “eat me” signal [61]. Consistent with our proteomic results, increased levels of ANXA1 have been previously noted in AD [62]. Preceding reports have assessed expression by microglia, astrocytes, and neurons [63, 64], but we have identified expression exclusively in astrocytes and neurons (Figure 6A–C). In AD, an accumulation of ANXA1 was predominantly observed in neurons with slight Tau deposits (Figure 6C, dashed line). In pathological conditions, astrocytes might express ANXA1 in an attempt to tag neurons “to be degraded” by microglia.

PRDX6 is an antioxidant enzyme, and its increased expression has been associated with astrocytes in AD [65]. Recently, a protective role of astrocytes via PRDX6 in Aβ proteostasis has been highlighted, since increased PRDX6 expression might mediate phagocytic activation of periplaque microglia [66]. Previous proteomic analysis in our laboratory revealed increased PRDX6 in the EC in AD, which was linked to microglia and astrocytes [29]. Here, we also identified an increase in PRDX6 levels in the AD amygdala. PRDX6 was associated with astrocytes in non‐AD and AD samples (Figure 7D–F, respectively), with remarkable colocalization with pathological markers (Figure 7E,F). In Aβ pathology, PRDX6 accumulation was specifically related to astrocytes in close contact with small plaques (Figure 7F, arrow), suggesting its involvement in Aβ proteostasis.

**FIGURE 9:**
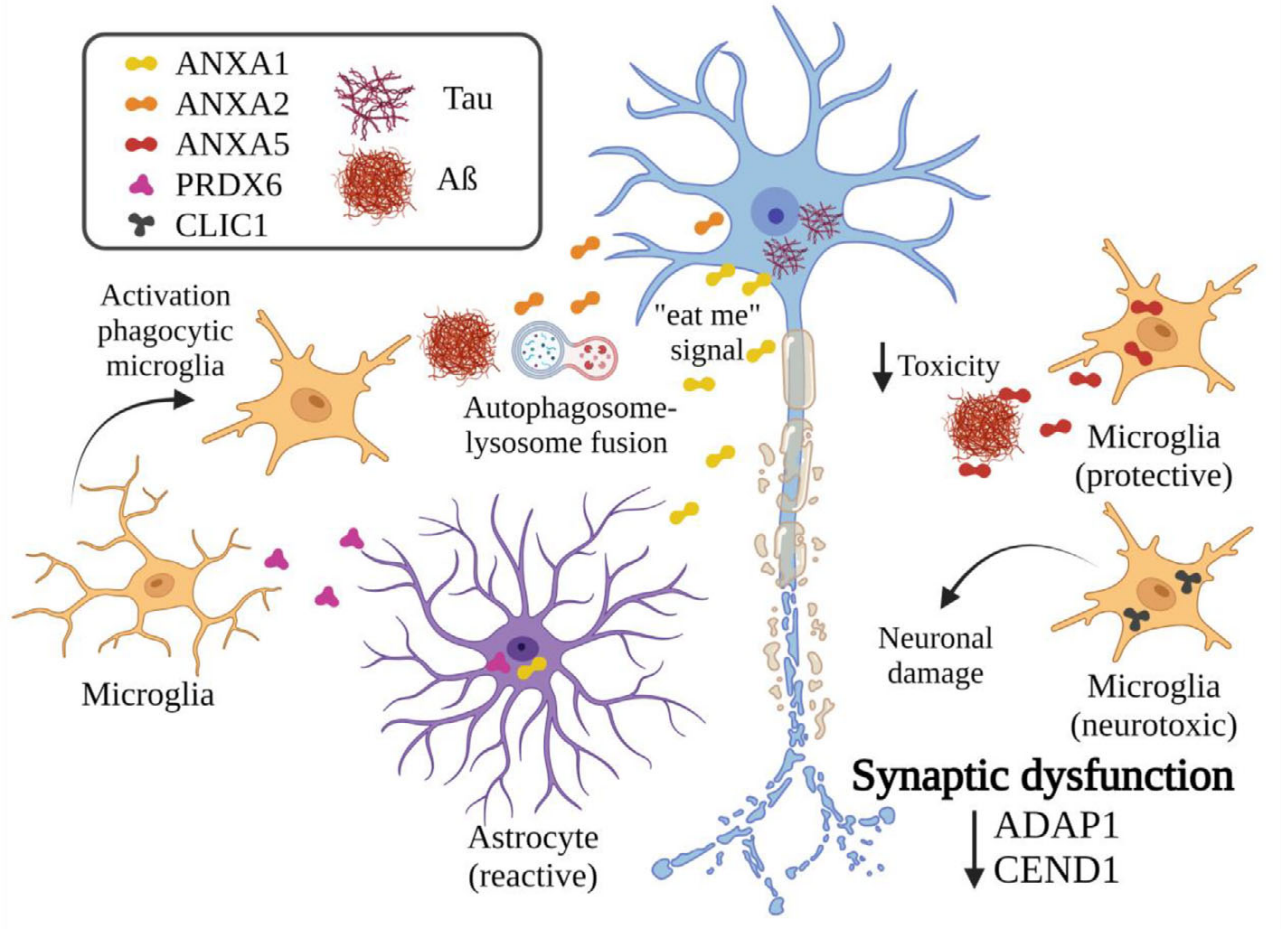
Synaptic and glial responses against injury. Representative scheme of neuronal and glial responses against pathology in the amygdala according to proteomic data analysis and the literature. Reductions in ADAP1 and CEND1 suggest synaptic dysfunction. To control the disease, ANXA2 might mediate autophagosome‐lysosome fusion. Astrocytes might promote the activation of phagocytic microglia (PRDX6) and mark neurons for their clearance by microglia (ANXA1). Microglia might have a dual role since protective (ANXA5) and neurotoxic (CLIC1) roles have been linked. Created with BioRender.com

Considering these results, AD pathology in the AC could cause synaptic dysfunction (ADAP1 and CEND1 reduction) accompanied by a glial response to damage. ANXA2 might mediate autophagosome‐lysosome fusion to contain the pathology. Astrocytes, via upregulated PRDX6 expression, might be mediating phagocytic microglia activation, as well as labeling neurons with ANXA1 for microglial degradation. However, microglia might have a dual role involving a protective function of ANXA5 in reducing pathology toxicity and a neurotoxic phenotype related to the increased CLIC1 expression that may promote neuronal damage (Figure 9).

To the best of our knowledge, this work comprises the first stereological analysis that includes volume and cell population estimations (employing specific cell markers), as well as pathology evaluation considering the same amygdaloid nuclei, facilitating the comprehension of the results. Furthermore, this study constitutes the first proteomic analysis of the human amygdala in AD. The combination of methodologies allowed us to elucidate the possible synaptic alterations as well as the potential participation of glial cells in response to pathology. Astrocytes might facilitate the protective actions of microglia, whereas microglia might play neuroprotective and neurotoxic roles. Moreover, the gradient observed in pathology distribution points out the relevance of the connections with olfactory areas and the hippocampal formation, suggesting a particular participation of the AC in AD.

## AUTHOR CONTRIBUTIONS

*Conceptualization*: Melania Gonzalez‐Rodriguez, Daniel Saiz‐Sanchez, Alino Martinez‐Marcos; *Methodology*: Melania Gonzalez‐Rodriguez, Sandra Villar‐Conde, Patricia Villanueva‐Anguita; *Formal analysis and investigation*: Melania Gonzalez‐Rodriguez; *Writing—original draft preparation*: Melania Gonzalez‐Rodriguez; *Writing—review and editing*: Sandra Villar‐Conde, Veronica Astillero‐Lopez, Isabel Ubeda‐Banon, Alicia Flores‐Cuadrado, Daniel Saiz‐Sanchez, Alino Martinez‐Marcos; *Funding acquisition*: Alino Martinez‐Marcos, Daniel Saiz‐Sanchez, Isabel Ubeda‐Banon; *Supervision*: Alino Martinez‐Marcos, Daniel Saiz‐Sanchez.

## FUNDING INFORMATION

The study was sponsored by the University of Castilla‐La Mancha/European Regional Development Fund (2021‐GRIN‐31233 to Alino Martinez–Marcos), Spanish Ministries of Economy and Competitiveness/European Regional Development Fund (grant no. SAF2016‐75768‐R to Alino Martinez‐Marcos) and Science and Innovation (grant no. PID2019‐108659RB‐I00 to Alino Martinez‐Marcos) and Autonomous Government of Castilla‐La Mancha/European Regional Development Fund (grant no. SBPLY/17/180501/000430 to Alino Martinez‐Marcos and Daniel Saiz‐Sanchez and SBPLY/21/180501/000093 to Alino Martinez‐Marcos and Isabel Ubeda‐Banon). Melania Gonzalez‐Rodriguez and Sandra Villar‐Conde held predoctoral fellowships granted by the University of Castilla‐La Mancha/European Social Fund.

## CONFLICT OF INTEREST STATEMENT

The authors declare that they have no conflict of interests.

## Supporting information


**Online Resource 1.** Antibodies detail.

**Online Resource 2.** LC‐MS/MS.

**Online Resource 3.** Volume data.

**Online Resource 4.** Increased astroglia density in the amygdaloid nuclei in AD. The MAP2‐positive cells/mm^3^ (a), Iba‐1‐positive cells/mm^3^ (b) and GFAP‐positive cells/mm^3^ (c) in the global AC and the different nuclei are shown (the graphs show the mean ± SEM, *p value <0.05, **p value <0.01, ***p value <0.001). AC, amygdaloid complex (Co, BLA); Co, cortical nucleus; BLA, basolateral complex (BM, BL, La); BM, basomedial nucleus; BL, basolateral nucleus; La, lateral nucleus.

**Online Resource 5.** MAP2 stereological quantification data.

**Online Resource 6.** Iba‐1 stereological quantification data.

**Online Resource 7.** GFAP stereological quantification data.

**Online Resource 8.** Aβ stereological quantification data.

**Online Resource 9.** Tau stereological quantification data.

**Online Resource 10.** Phosphorylated TDP‐43 (TDP‐43‐P) deposits in amygdala in AD. (a) Immunohistochemistry against TDP‐43‐P in AC in AD (case 2). Note different deposition pattern between nuclei. (b) Abundant cellular accumulations of TDP‐43‐P can be observed in Co, while in (c) BM and (d) BLTDP‐43‐P appears in clusters. In contrast, (e) scarce deposits are in La. Co: Cortical nucleus, BM: Basomedial nucleus, BL: Basolateral nucleus, La: Lateral nucleus, EC: Entorhinal cortex. Scale bar = 1000 μm in (a); and 50 μm in (b,c,d,e).

**Online Resource 11.** (a) Protein lists that interact with APP or MAPT, and preferentially expressed in neurons, microglia and astrocytes. (b) SynGo analysis from 178 DEP (dataset version: 20210225). (c) Metascape analysis of 178 DEP.

**Online Resource 12.** *Proteomic analysis*. Volcano plot (a) showed 108 up‐(green) and 70 downregulated (red) DEPs (FC > 1.5, *p* value <0.05). Venn diagrams identified that are present in the different cellular types, neurons (b), microglia (c) and astrocytes (d), and interact with pathological proteinsin AD. Cellular responses to stress, regulation of proteolysis and response to wounding were exposed as impaired processes by functional analysis with Metascape (f). DEPs: differentially expressed proteins.

## Data Availability

All data generated or analyzed during this study are included in this published article (and its additional files). The mass spectrometry proteomics data have been deposited to the ProteomeXchange Consortium via the PRIDE [39] partner repository with the dataset identifier PXD038322.
