# Supplementary material for: Human amygdala involvement in Alzheimer's disease revealed by stereological and dia‐PASEF analysis
Source: Brain Pathol. 2023 Jun 18;33(5):e13180. doi: 10.1111/bpa.13180 (PMC10467039; doi:10.1111/bpa.13180)
Supplement: Supplementary file 1 — Online Resource 1. Antibodies detail. [file BPA-33-e13180-s008.pdf]

Online Resource 1. Antibodies detail.

Antigen	Antibody	Manufacturer	Cat. Nº	Dilution	Blocking buffer	Antibody dilution buffer
**Immunohistochemistry**						
**MAP2**	Mouse anti-MAP2	Invitrogen	13-1500	1:500	PBS- 0.4% Tx100- 3% NHS	PBS- 0.4% Tx100- 3% NHS
**Iba-1**	Rabbit anti-Iba-1	Wako	019-19741	1:2,000	PBS- 0.1% Tx100	PBS- 0.1% Tx100
**GFAP**	Rabbit anti-GFAP	Dako	Z0334	1:10,000	PBS- 0.1% Tx100- 10% NHS	PBS- 0.1% Tx100- 10% NHS
**Aβ**	Mouse anti- β- amyloid	Merck	05-831-I	1:5,000	PBS- 0.3% Tx100- 5% NDS	PBS- 0.3% Tx100
**Tau**	Mouse anti- Tau	Cell Signaling	4019	1:1600	PBS- 0.3% Tx100- 5%NDS	PBS- 0.3% Tx100
**TDP-43-P**	Rabbit anti- phosphorylated-TDP-43	Invitrogen	PA5-114661	1:100	PBS- 0.3% Tx100- 5%NDS	PBS- 0.3% Tx100- 5%NDS
**Immunofluorescence**						
**MAP2**	Mouse anti-MAP2	Invitrogen	13-1500	1:100	TBS- 0.3% Tx100- 10% NDS	TBS- 0.3% Tx100- 10% NDS
**Iba-1**	Goat anti- Iba-1	Abcam	ab5076	1:1,000	TBS- 0.3% Tx100- 10% NDS	TBS- 0.3% Tx100- 10% NDS
**GFAP**	Goat anti- GFAP	Abcam	ab53554	1:500	TBS- 0.3% Tx100- 10% NDS	TBS- 0.3% Tx100- 10% NDS
**Aβ**	Mouse anti- β- amyloid	Merck	05-831-I	1:1,000	TBS- 0.3% Tx100- 10% NDS	TBS- 0.3% Tx100- 10% NDS
**Aβ**	Rabbit anti- β- amyloid	Cell Signaling	2454	1:250	TBS- 0.3% Tx100- 10% NDS	TBS- 0.3% Tx100- 10% NDS
**Tau**	Mouse anti- Tau	Cell Signaling	4019	1:800	TBS- 0.3% Tx100- 10% NDS	TBS- 0.3% Tx100- 10% NDS
**Tau**	Rabbit anti-Tau	Cell Signaling	46687	1:100	TBS- 0.3% Tx100- 10% NDS	TBS- 0.3% Tx100- 10% NDS
**ADAP1**	Goat anti- Centaurin alpha 1	Abcam	ab27476	1:50	TBS- 0.3% Tx100- 10% NDS	TBS- 0.3% Tx100- 10% NDS
**CEND1**	Rabbit anti- CEND1	Abcam	ab113076	1:250	TBS- 0.3% Tx100- 10% NDS	TBS- 0.3% Tx100- 10% NDS
**ANXA1**	Rabbit anti- Annexin A1	Invitrogen	71-3400	1:50	TBS- 0.3% Tx100- 10% NDS	TBS- 0.3% Tx100- 10% NDS
**ANXA2**	Rabbit anti- Annexin A2	Abcam	ab32571	1:100	TBS- 0.3% Tx100- 10% NDS	TBS- 0.3% Tx100
**ANXA5**	Mouse anti- Annexin A5	Santa Cruz	sc-74438	1:50	TBS- 0.3% Tx100- 10% NDS	TBS- 0.3% Tx100- 5% NDS
**CLIC1**	Rabbit anti- CLICL1	Abcam	ab229917	1:50	TBS- 0.3% Tx100- 10% NDS	TBS- 0.3% Tx100- 10% NDS
**PRDX6**	Rabbit anti-PRDX6	Abcam	ab59543	1:100	TBS+0.3% TX-100 +10% NDS	TBS- 0.3% Tx100
