# Supplementary material for: Human amygdala involvement in Alzheimer's disease revealed by stereological and dia‐PASEF analysis
Source: Brain Pathol. 2023 Jun 18;33(5):e13180. doi: 10.1111/bpa.13180 (PMC10467039; doi:10.1111/bpa.13180)
Supplement: Supplementary file 2 — Online Resource 2. LC‐MS/MS. [file BPA-33-e13180-s005.pdf]

LC-MS/MS

Samples were separated with the predefined 60 sample per day method (21 min gradient time, 200 ng peptides), on a reverse-phase analytical column 8 cm x 150 µm, C18 1.5 µm (EV1109) heated to 40 ºC. Mobile phases were H_2O and acetonitrile, buffered with 0.1% formic acid (LC-MS grade, Thermo Fisher Scientific).

The acquisition modes recording spectra from 475 to 1,000 m/z. Ion mobility resolution was calibrated with three Agilent ESI-L Tuning Mix ions (mass spectra, ion mobility: 622.0289 m/z, 0.9848 Vs cm−2; 922.0097 m/z, 1.1895 Vs cm−2; 1221.9906 m/z, 1.3820 Vs cm−2) and set to 0.85-1.30 Vs/cm−2. DdaPASEF consisted of four PASEF MS/MS. The accumulation of ions and the ramp times parallel to the scans were 100 ms, with a total cycle time of 0.53 s. Any individually charged ions were excluded from MS/MS analysis. Moreover, precursors that reached the target value of 20,000 were excluded for 0.4 min. A Q window of ± 2 Da for m/z <700 and ± 3 Da for m/z >800 was used for isolating precursors. The 12 most intense ions were fragmented. For diaPASEF, 21 non-overlapping windows of 25 Da were set, making a total cycle time of 0.95 s. The collision energy was decreased linearly from 45 eV at ion mobility of 1.30 Vs cm−2 to 27 eV at 0.85 Vs cm−2 in both ddaPASEF and diaPASEF methods.
