# Supplementary material for: Human amygdala involvement in Alzheimer's disease revealed by stereological and dia‐PASEF analysis
Source: Brain Pathol. 2023 Jun 18;33(5):e13180. doi: 10.1111/bpa.13180 (PMC10467039; doi:10.1111/bpa.13180)
Supplement: Supplementary file 3 — Online Resource 3. Volume data. [file BPA-33-e13180-s004.pdf]

**Online Resource 3. Volume data.**

**Table a.** Estimated amygdaloid complex volume.

| | *Coefficient of Error (Gundersen). m=1* | *Sections* | *Grid Size (µm)* | *Volume Corrected for OverProjection ($mm^3$)* |
|---|---|---|---|---|
| ***AD*** | | | | |
| *1* | 0.024 | 4 | 500 | 230.01 |
| *2* | 0.022 | 4 | 500 | 175.18 |
| *3* | 0.025 | 4 | 500 | 172.33 |
| *4* | 0.03 | 3 | 500 | 235.89 |
| *5* | 0.022 | 4 | 500 | 257.09 |
| *6* | 0.023 | 4 | 500 | 180.03 |
| *7* | 0.021 | 4 | 250 | 234.13 |
| *8* | 0.023 | 4 | 500 | 218.10 |
| *9* | 0.025 | 4 | 500 | 215.80 |
| *10* | 0.023 | 4 | 500 | 210.538 |
| ***Non-AD*** | | | | |
| *19* | 0.021 | 4 | 500 | 277.46 |
| *20* | 0.023 | 4 | 500 | 404.43 |
| *21* | 0.021 | 4 | 500 | 273.11 |
| *22* | 0.024 | 4 | 500 | 253.11 |
| *23* | 0.023 | 4 | 500 | 274.73 |
| *24* | 0.022 | 4 | 250 | 267.76 |
| *25* | 0.024 | 4 | 500 | 249.23 |
| *26* | 0.023 | 4 | 500 | 276.03 |
| *27* | 0.024 | 4 | 500 | 232.99 |
| *28* | 0.023 | 4 | 500 | 281.98 |

**Table b.** Estimated cortical nucleus volume.

| | ***Coefficient of Error (Gundersen). m=1*** | ***Sections*** | ***Grid Size (μm)*** | ***Volume Corrected for OverProjection ($mm^3$)*** |
|---|---|---|---|---|
| ***AD*** | | | | |
| *1* | 0.026 | 4 | 500 | 34.34 |
| *2* | 0.032 | 4 | 500 | 17.16 |
| *3* | 0.032 | 4 | 500 | 25.66 |
| *4* | 0.034 | 3 | 500 | 19.25 |
| *5* | 0.025 | 4 | 500 | 32.78 |
| *6* | 0.031 | 4 | 500 | 17.84 |
| *7* | 0.025 | 4 | 250 | 18.23 |
| *8* | 0.03 | 4 | 500 | 24.76 |
| *9* | 0.032 | 4 | 500 | 21.71 |
| *10* | 0.029 | 4 | 500 | 21.91 |
| ***Non-AD*** | | | | |
| *19* | 0.032 | 3 | 500 | 31.76 |
| *20* | 0.026 | 4 | 500 | 38.66 |
| *21* | 0.026 | 4 | 500 | 37.58 |
| *22* | 0.025 | 4 | 500 | 33.43 |
| *23* | 0.027 | 4 | 500 | 22.44 |
| *24* | 0.019 | 4 | 250 | 32.02 |
| *25* | 0.024 | 4 | 500 | 31.78 |
| *26* | 0.032 | 4 | 500 | 19.13 |
| *27* | 0.027 | 4 | 500 | 26.41 |
| *28* | 0.024 | 4 | 250 | 31.39 |

**Table c.** Estimated basolateral complex volume.

| | *Coefficient of Error (Gundersen). m=1* | *Sections* | *Grid Size (μm)* | *Volume Corrected for OverProjection ($mm^3$)* |
|---|---|---|---|---|
| ***AD*** | | | | |
| *1* | 0.025 | 4 | 500 | 192.46 |
| *2* | 0.022 | 4 | 500 | 154.61 |
| *3* | 0.025 | 4 | 500 | 144.15 |
| *4* | 0.03 | 3 | 500 | 213.30 |
| *5* | 0.023 | 4 | 500 | 219.19 |
| *6* | 0.023 | 4 | 500 | 158.06 |
| *7* | 0.021 | 4 | 250 | 212.95 |
| *8* | 0.023 | 4 | 500 | 188.53 |
| *9* | 0.025 | 4 | 500 | 189.79 |
| *10* | 0.023 | 4 | 500 | 185.44 |
| ***Non-AD*** | | | | |
| *19* | 0.03 | 4 | 500 | 237.54 |
| *20* | 0.023 | 4 | 500 | 358.55 |
| *21* | 0.021 | 4 | 500 | 230.73 |
| *22* | 0.025 | 4 | 500 | 216.93 |
| *23* | 0.024 | 4 | 500 | 249.69 |
| *24* | 0.023 | 4 | 250 | 231.58 |
| *25* | 0.025 | 4 | 500 | 214.95 |
| *26* | 0.023 | 4 | 500 | 254.38 |
| *27* | 0.024 | 4 | 500 | 201.79 |
| *28* | 0.023 | 4 | 250 | 247.02 |

**Table d.** Estimated basomedial nucleus volume.

| | *Coefficient of Error (Gundersen). m=1* | *Sections* | *Grid Size (μm)* | *Volume Corrected for OverProjection ($mm^3$)* |
|---|---|---|---|---|
| ***AD*** | | | | |
| *1* | 0.025 | 4 | 500 | 42.21 |
| *2* | 0.036 | 4 | 500 | 11.75 |
| *3* | 0.032 | 4 | 500 | 15.69 |
| *4* | 0.041 | 3 | 500 | 25.59 |
| *5* | 0.027 | 4 | 500 | 30.05 |
| *6* | 0.041 | 4 | 500 | 10.51 |
| *7* | 0.028 | 4 | 250 | 11.32 |
| *8* | 0.033 | 4 | 500 | 15.89 |
| *9* | 0.036 | 4 | 500 | 19.03 |
| *10* | 0.034 | 4 | 500 | 16.61 |
| ***Non-AD*** | | | | |
| *19* | 0.033 | 4 | 500 | 15.90 |
| *20* | 0.028 | 4 | 500 | 39.08 |
| *21* | 0.023 | 4 | 500 | 49.66 |
| *22* | 0.024 | 4 | 500 | 44.68 |
| *23* | 0.033 | 4 | 500 | 20.15 |
| *24* | 0.021 | 4 | 250 | 16.45 |
| *25* | 0.03 | 4 | 500 | 23.68 |
| *26* | 0.029 | 4 | 500 | 21.15 |
| *27* | 0.031 | 4 | 500 | 15.65 |
| *28* | 0.026 | 4 | 250 | 20.21 |

**Table e.** Estimated basolateral nucleus volume.

| | ***Coefficient of Error (Gundersen). m=1*** | ***Sections*** | ***Grid Size (µm)*** | ***Volume Corrected for OverProjection (mm$^3$)*** |
|---|---|---|---|---|
| ***AD*** | | | | |
| *1* | 0.026 | 4 | 500 | 80.59 |
| *2* | 0.026 | 4 | 500 | 49.41 |
| *3* | 0.027 | 4 | 500 | 57.80 |
| *4* | 0.03 | 3 | 500 | 83.00 |
| *5* | 0.022 | 4 | 500 | 87.01 |
| *6* | 0.031 | 4 | 500 | 30.31 |
| *7* | 0.026 | 4 | 250 | 54.10 |
| *8* | 0.028 | 4 | 500 | 58.90 |
| *9* | 0.027 | 4 | 500 | 69.63 |
| *10* | 0.027 | 4 | 500 | 56.08 |
| ***Non-AD*** | | | | |
| *19* | 0.026 | 4 | 500 | 77.33 |
| *20* | 0.024 | 4 | 500 | 113.80 |
| *21* | 0.025 | 4 | 500 | 76.90 |
| *22* | 0.024 | 4 | 500 | 69.90 |
| *23* | 0.025 | 4 | 500 | 81.05 |
| *24* | 0.025 | 4 | 250 | 68.93 |
| *25* | 0.024 | 4 | 500 | 67.46 |
| *26* | 0.029 | 4 | 500 | 70.61 |
| *27* | 0.032 | 4 | 500 | 62.23 |
| *28* | 0.022 | 4 | 250 | 90.19 |

**Table f.** Estimated lateral nucleus volume.

| | *Coefficient of Error (Gundersen). m=1* | *Sections* | *Grid Size (µm)* | *Volume Corrected for OverProjection ($mm^3$)* |
|---|---|---|---|---|
| ***AD*** | | | | |
| *1* | 0.03 | 4 | 500 | 66.64 |
| *2* | 0.023 | 4 | 500 | 89.64 |
| *3* | 0.027 | 4 | 500 | 66.93 |
| *4* | 0.03 | 3 | 500 | 99.89 |
| *5* | 0.026 | 4 | 500 | 97.48 |
| *6* | 0.023 | 4 | 500 | 113.09 |
| *7* | 0.02 | 4 | 250 | 143.14 |
| *8* | 0.022 | 4 | 500 | 108.98 |
| *9* | 0.025 | 4 | 500 | 96.18 |
| *10* | 0.023 | 4 | 500 | 109.55 |
| ***Non-AD*** | | | | |
| *19* | 0.024 | 4 | 500 | 137.09 |
| *20* | 0.023 | 4 | 500 | 194.80 |
| *21* | 0.02 | 4 | 500 | 100.03 |
| *22* | 0.029 | 4 | 500 | 98.83 |
| *23* | 0.024 | 4 | 500 | 141.81 |
| *24* | 0.022 | 4 | 250 | 141.02 |
| *25* | 0.027 | 4 | 500 | 119.01 |
| *26* | 0.022 | 4 | 500 | 157.09 |
| *27* | 0.022 | 4 | 500 | 118.85 |
| *28* | 0.026 | 4 | 250 | 129.43 |
