# Supplementary material for: Human amygdala involvement in Alzheimer's disease revealed by stereological and dia‐PASEF analysis
Source: Brain Pathol. 2023 Jun 18;33(5):e13180. doi: 10.1111/bpa.13180 (PMC10467039; doi:10.1111/bpa.13180)
Supplement: Supplementary file 4 — Online Resource 4. Increased astroglia density in the amygdaloid nuclei in AD. The MAP2‐positive cells/mm3 (a), Iba‐1‐positive cells/mm3 (b) and GFAP‐positive cells/mm3 (c) in the global AC and the different nuclei are shown (the graphs show the mean ± SEM, *p value <0.05, **p value <0.01, ***p value <0.001). AC, amygdaloid complex (Co, BLA); Co, cortical nucleus; BLA, basolateral complex (BM, BL, La); BM, basomedial nucleus; BL, basolateral nucleus; La, lateral nucleus. [file BPA-33-e13180-s006.pdf]

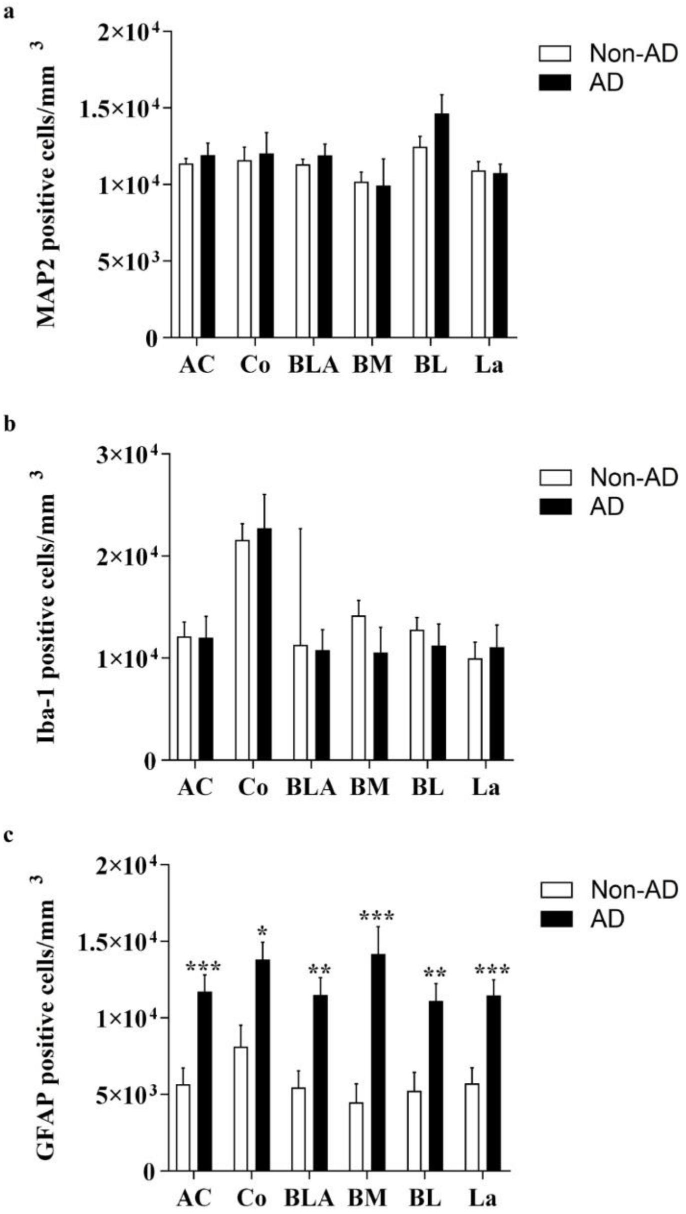

Online Resource 4 *Increased astroglia density in the amygdaloid nuclei in AD.* The MAP2-positive cells/mm^3 (a), Iba-1-positive cells/mm^3 (b) and GFAP-positive cells/mm^3 (c) in the global AC and the different nuclei are shown (the graphs show the mean ± SEM, * p value < 0.05, ** p value < 0.01, *** p value < 0.001). AC: Amygdaloid complex (Co, BLA), Co: Cortical nucleus, BLA: Basolateral complex (BM, BL, La), BM: Basomedial nucleus, BL: Basolateral nucleus, La: Lateral nucleus.
