# Supplementary material for: Human amygdala involvement in Alzheimer's disease revealed by stereological and dia‐PASEF analysis
Source: Brain Pathol. 2023 Jun 18;33(5):e13180. doi: 10.1111/bpa.13180 (PMC10467039; doi:10.1111/bpa.13180)
Supplement: Supplementary file 5 — Online Resource 5. MAP2 stereological quantification data. [file BPA-33-e13180-s003.pdf]

Online Resource 5. MAP2 stereological quantification data.

Table a. Cortical nucleus MAP2 stereological quantification data.

	Total Markers Counted	*Number of Sections*	*Number of Sampling Sites*	*Coefficient of Error (Gundersen). m=1*	*Counting Frame Area (XY) (µm²)*	*Sampling Grid Area (XY) (µm²)*	*Estimated Population using Mean Section Thickness with Counts*	*Measured Volume (mm³)*	*Numerical Density (cell/mm³)*
AD									
1	112	4	88	0.1	2500	422500	394694.53	24.28	16254.01
2	108	4	288	0.1	2500	90000	73292.08	15.52	4721.55
3	152	4	143	0.08	2500	250000	289765.19	21.70	13350.28
4	129	3	110	0.09	2500	250000	247576.38	17.57	14093.99
5	124	4	96	0.09	2500	490000	444362.19	30.25	14691.75
6	150	4	97	0.08	2500	250000	293173.09	15.26	19192.62
7	157	4	273	0.08	2500	90000	110737.77	15.24	7267.07
8	113	4	137	0.1	2500	250000	221105.17	20.80	10630.01
9	101	3	147	0.1	2500	160000	130745.18	14.21	9201.38
10	167	4	190	0.08	2500	160000	205463.66	19.07	10775.71
Non-AD									
19	116	3	145	0.1	2500	250000	213860.66	22.67	9439.18
20	101	4	123	0.1	2500	360000	302574.28	26.34	11489.17
21	116	4	106	0.1	2500	490000	430013.44	31.27	13753.65
22	103	4	81	0.1	2500	490000	418217.88	23.52	17781.45
23	110	3	123	0.1	2500	202500	189030.59	15.76	11992.35
24	139	3	135	0.09	2500	250000	259529.88	21.17	12260.25
25	131	4	146	0.09	2500	302500	283811.31	27.61	10278.62
26	155	4	178	0.09	2500	160000	186132.25	17.59	10583.09
27	125	4	145	0.09	2500	250000	240961.75	23.29	10347.92
28	144	4	212	0.09	2500	160000	170364.2	21.12	8065.57

Table b. Basomedial nucleus MAP2 stereological quantification data.

	Total Markers Counted	Number of Sections	Number of Sampling Sites	Coefficient of Error (Gundersen). m=1	Counting Frame Area (XY) (µm²)	Sampling Grid Area (XY) (µm²)	Estimated Population using Mean Section Thickness with Counts	Measured Volume (mm³)	Numerical Density (cell/mm³)
**AD**									
1	111	4	126	0.1	2500	422500	385302.28	32.37	11903.32
2	119	4	386	0.09	2500	40000	35245.73	9.45	3729.94
3	130	4	192	0.09	2500	122500	123984.94	14.65	8463.71
4	118	3	167	0.1	2500	202500	178202.28	21.57	8261.04
5	110	4	177	0.1	2500	250000	203860.36	27.31	7464.35
6	163	4	87	0.1	2500	202500	255476	10.51	24315.76
7	119	4	175	0.09	2500	90000	84338.17	9.56	8818.94
8	123	4	174	0.09	2500	160000	152522.8	17.09	8922.44
9	113	4	197	0.1	2500	160000	140746.66	19.23	7318.89
10	155	4	188	0.08	2500	122500	145845.09	14.17	10295.58
**Non-AD**									
19	135	3	113	0.09	2500	160000	164289.08	11.31	14523.048
20	109	4	131	0.1	2500	422500	374113.41	35.96	10404.79
21	143	4	186	0.09	2500	360000	394873.69	41.35	9548.71
22	100	4	134	0.1	2500	422500	346532.41	36.43	9513.61
23	133	3	193	0.09	2500	90000	102527.88	10.87	9432.88
24	114	3	125	0.1	2500	160000	135421.84	12.64	10717.91
25	162	4	171	0.08	2500	202500	236446.69	21.77	10859.98
26	108	4	133	0.1	2500	250000	212750.58	20.14	10564.27
27	111	4	145	0.1	2500	160000	136084.52	13.91	9782.58
28	142	4	261	0.09	2500	90000	95347.59	14.64	6513.21

Table c. Basolateral nucleus MAP2 stereological quantification data.

	Total Markers Counted	*Number of Sections*	*Number of Sampling Sites*	*Coefficient of Error (Gundersen). m=1*	*Counting Frame Area (XY) (μm²)*	*Sampling Grid Area (XY) (μm²)*	*Estimated Population using Mean Section Thickness with Counts*	*Measured Volume (mm³)*	*Numerical Density (cell/mm³)*
AD									
1	145	4	131	0.09	2500	810000	985157.5	66.45	14826.06
2	104	4	138	0.1	2500	490000	377298.13	42.7	8835.69
3	171	4	105	0.08	2500	810000	1069707.5	52.85	20241.63
4	199	3	190	0.08	2500	640000	944027.19	77.74	12142.81
5	204	4	145	0.07	2500	810000	1276813.38	74.42	17157.07
6	244	4	153	0.07	2500	360000	697555.56	34.09	20463.02
7	132	4	127	0.09	2500	640000	688230.31	50.27	13690.16
8	129	4	140	0.09	2500	640000	650518.75	58.19	11180.03
9	152	4	158	0.08	2500	640000	786815.81	64.31	12235.46
10	170	4	135	0.08	2500	640000	847953.94	54.02	15696.19
Non-AD									
19	129	4	127	0.09	2500	902500	907650.63	72.42	12533.5
20	157	4	159	0.08	2500	1000000	1304295.5	100.78	12941.75
21	152	4	174	0.08	2500	640000	764007.13	70.72	10803.42
22	144	4	148	0.08	2500	640000	760779.69	58.17	13078.27
23	164	4	205	0.08	2500	640000	909977.06	82.43	11039.5
24	136	3	94	0.09	2500	810000	819915.38	47.97	17091.22
25	136	4	157	0.09	2500	640000	652387.31	62.07	10510.48
26	140	4	172	0.09	2500	640000	715445.88	71.23	10044.39
27	147	4	135	0.09	2500	640000	723738.19	52.94	13671.87
28	181	4	167	0.08	2500	490000	678444.38	51.82	13093.14

Table d. Lateral nucleus MAP2 stereological quantification data.

	Total Markers Counted	*Number of Sections*	*Number of Sampling Sites*	*Coefficient of Error (Gundersen). m=1*	*Counting Frame Area (XY) (μm²)*	*Sampling Grid Area (XY) (μm²)*	*Estimated Population using Mean Section Thickness with Counts*	*Measured Volume (mm³)*	*Numerical Density (cell/mm³)*
AD									
1	202	4	194	0.08	2500	490000	826188.75	61.36	13465.54
2	103	4	159	0.1	2500	810000	637139.63	80.46	7918.76
3	110	4	127	0.1	2500	810000	719345.13	64.67	11123.7
4	119	3	135	0.1	2500	1000000	883924.75	87.37	10117.47
5	126	4	131	0.09	2500	1000000	936630.38	81.22	11531.59
6	173	4	178	0.08	2500	1000000	1377568.88	113.18	12171.49
7	165	4	230	0.08	2500	810000	1064196.5	118.94	8947.11
8	149	4	164	0.08	2500	1000000	1136516.38	104.83	10841.62
9	187	4	300	0.08	2500	490000	781073.25	92.77	8419.24
10	160	4	154	0.09	2500	1000000	1247521.63	97.44	12802.6
Non-AD									
19	150	4	229	0.08	2500	1000000	1176397.63	144.75	8126.93
20	154	4	185	0.08	2500	1440000	1795137.75	166.66	10771.13
21	117	4	156	0.09	2500	810000	730959.75	79.35	9211.72
22	138	3	143	0.09	2500	640000	701847.75	58.54	11988.48
23	162	4	219	0.08	2500	1000000	1406385.25	140.31	10023.27
24	111	3	136	0.1	2500	1440000	1010442.56	99.94	10110.97
25	107	4	125	0.1	2500	1440000	1046146.38	93.77	11155.97
26	113	4	99	0.1	2500	2560000	2371849.75	161.75	14663.68
27	183	4	188	0.08	2500	810000	1126587.5	98.89	11392.81
28	141	4	150	0.09	2500	1000000	1119092.25	94.77	11809.11
