# Supplementary material for: Human amygdala involvement in Alzheimer's disease revealed by stereological and dia‐PASEF analysis
Source: Brain Pathol. 2023 Jun 18;33(5):e13180. doi: 10.1111/bpa.13180 (PMC10467039; doi:10.1111/bpa.13180)
Supplement: Supplementary file 6 — Online Resource 6. Iba‐1 stereological quantification data. [file BPA-33-e13180-s001.pdf]

Online Resource 6. Iba-1 stereological quantification data.

Table a. Cortical nucleus Iba-1 stereological quantification data.

	Total Markers Counted	*Number of Sections*	*Number of Sampling Sites*	*Coefficient of Error (Gundersen). m=1*	*Counting Frame Area (XY) (μm²)*	*Sampling Grid Area (XY) (μm²)*	*Estimated Population using Mean Section Thickness with Counts*	*Measured Volume (mm³)*	*Numerical Density (cell/mm³)*
AD									
1	173	4	88	0.08	2500	490000	736846.50	27.13	27162.54
2	132	4	151	0.09	2500	160000	159178.75	14.20	11212.30
3	143	4	148	0.09	2500	250000	269946.19	23.17	11649.68
4	145	3	69	0.08	2500	490000	542024.00	20.99	25823.83
5	178	4	98	0.08	2500	490000	665370.50	30.43	21867.05
6	129	4	58	0.09	2500	490000	514952.00	18.01	28592.72
7	255	4	146	0.07	2500	160000	371721.81	14.53	25587.46
8	192	4	68	0.07	2500	490000	727029.56	20.35	35721.88
9	147	3	400	0.09	2500	62500	69674.70	15.38	4529.07
10	182	4	66	0.08	2500	490000	701052.75	19.97	35112.50
Non-AD									
20	153	4	114	0.08	2500	490000	578772.13	33.84	17101.02
21	146	3	77	0.09	2500	490000	516282.63	22.74	22702.92
22	133	3	65	0.1	2500	490000	458712.38	19.52	23502.26
23	107	4	64	0.1	2500	490000	402713.75	19.81	20329.84
24	127	4	77	0.09	2500	490000	461251.88	23.26	19831.88
25	174	4	90	0.08	2500	490000	676215.63	26.83	25203.71
26	133	4	51	0.09	2500	640000	640521.75	20.84	30737.42
27	98	4	82	0.1	2500	490000	347504.66	23.96	14504.32
28	139	4	90	0.09	2500	490000	572333.44	28.02	20425.38

Table b. Basomedial nucleus Iba-1 stereological quantification data.

	Total Markers Counted	Number of Sections	Number of Sampling Sites	Coefficient of Error (Gundersen). m=1	Counting Frame Area (XY) (μm²)	Sampling Grid Area (XY) (μm²)	Estimated Population using Mean Section Thickness with Counts	Measured Volume (mm³)	Numerical Density (cell/mm³)
AD									
1	204	4	119	0.07	2500	490000	859130.00	36.76	23374.51
2	65	4	402	0.12	2500	62500	38073.43	9.84	3867.51
3	145	4	393	0.09	2500	62500	67242.44	15.08	4459.23
4	129	4	176	0.09	2500	250000	237810.94	27.76	8566.55
5	210	4	280	0.07	2500	160000	247972.97	28.42	8726.83
6	117	4	105	0.1	2500	160000	160599.59	10.07	15949.27
7	230	4	419	0.07	2500	40000	79469.2	10.10	7870.97
8	102	4	131	0.1	2500	160000	119450.83	13.18	9064.76
9	50	4	672	0.14	2500	40000	14935.56	16.68	895.37
10	251	4	143	0.07	2500	160000	321709.19	14.01	22969.38
Non-AD									
19	177	3	348	0.08	2500	40000	49423.49	8.51	5805.81
20	161	4	153	0.08	2500	360000	447020.19	34.26	13048.37
21	157	3	126	0.08	2500	360000	411102.75	28.73	14311.77
22	319	4	242	0.06	2500	250000	666664.44	37.82	17629.26
23	193	4	175	0.08	2500	160000	236671.14	17.59	13454.95
24	165	4	167	0.08	2500	160000	202121.47	16.19	12481.64
25	356	4	200	0.06	2500	160000	454472.88	20.08	22636.16
26	113	4	73	0.1	2500	490000	404133.38	21.54	18765.57
27	210	4	244	0.07	2500	90000	136548.30	13.66	9999.44
28	202	4	190	0.08	2500	160000	260677.58	19.09	13659.13

Table c. Basolateral nucleus Iba-1 stereological quantification data.

	Total Markers Counted	*Number of Sections*	*Number of Sampling Sites*	*Coefficient of Error (Gundersen). m=1*	*Counting Frame Area (XY) (µm²)*	*Sampling Grid Area (XY) (µm²)*	*Estimated Population using Mean Section Thickness with Counts*	*Measured Volume (mm³)*	*Numerical Density (cell/mm³)*
AD									
1	227	4	162	0.07	2500	640000	1292839.75	66.96	19308.36
2	101	4	450	0.1	2500	160000	119363.00	45.76	2608.56
3	179	4	236	0.08	2500	360000	512909.00	54.39	9431.44
4	165	4	310	0.08	2500	490000	589492.75	96.43	6113.21
5	212	4	195	0.07	2500	640000	985542.88	77.13	12777.52
6	96	4	79	0.1	2500	640000	518432.44	31.39	16518.90
7	164	4	215	0.08	2500	360000	516745.75	49.13	10519.08
8	149	4	119	0.08	2500	640000	718690.31	47.97	14983.20
9	82	4	1565	0.12	2500	62500	39781.09	61.18	650.24
10	186	4	119	0.07	2500	640000	944113.63	48.51	19462.73
Non-AD									
19	99	4	197	0.1	2500	490000	342714.19	59.44	5766.06
20	170	4	158	0.08	2500	1000000	1301878.50	103.46	12583.89
21	147	4	139	0.09	2500	810000	848052.19	71.47	11865.28
22	167	4	155	0.08	2500	640000	901250.69	62.69	14375.92
23	170	4	186	0.08	2500	640000	836201.94	75.90	11016.86
24	181	4	164	0.08	2500	640000	922348.19	65.75	14027.98
25	231	4	139	0.07	2500	640000	1207363.88	56.95	21200.42
26	185	4	182	0.08	2500	640000	862383.25	71.49	12062.45
27	132	4	137	0.09	2500	640000	629054.56	55.16	11405.05
28	189	4	194	0.08	2500	640000	1046232.81	77.09	13572.37

Table d. Lateral nucleus Iba-1 stereological quantification data.

	Total Markers Counted	*Number of Sections*	*Number of Sampling Sites*	*Coefficient of Error (Gundersen). m=1*	*Counting Frame Area (XY) (µm²)*	*Sampling Grid Area (XY) (µm²)*	*Estimated Population using Mean Section Thickness with Counts*	*Measured Volume (mm³)*	*Numerical Density (cell/mm³)*
AD									
1	355	4	199	0.06	2500	490000	1490826.25	63.43	23503.93
2	130	4	272	0.09	2500	490000	479929.84	85.46	5615.78
3	119	4	185	0.1	2500	490000	499261.28	58.33	8559.86
4	179	4	183	0.08	2500	1000000	1287462.25	115.00	11195.62
5	280	4	175	0.06	2500	810000	1655242.13	90.48	18294.44
6	147	4	224	0.09	2500	810000	1035313.19	113.39	9130.79
7	159	4	247	0.08	2500	810000	1181195.13	127.62	9255.27
8	167	4	294	0.08	2500	490000	653026.13	90.35	7228.06
9	21	4	2174	0.23	2500	62500	10775.45	86.11	125.14
10	243	4	187	0.07	2500	810000	1716095.25	97.34	17629.13
Non-AD									
19	116	4	585	0.1	2500	360000	291832.94	133.64	2183.69
20	289	4	310	0.06	2500	810000	1816474.00	159.90	11360.06
21	127	4	177	0.09	2500	810000	730847.75	91.42	7994.84
22	183	4	158	0.08	2500	810000	1251328.50	84.08	14883.36
23	223	4	244	0.07	2500	810000	1366829.88	125.01	10934.11
24	284	4	262	0.06	2500	810000	1863937.38	135.36	13770.73
25	227	4	190	0.07	2500	810000	1562544.50	98.03	15939.40
26	115	4	664	0.09	2500	360000	307634.94	153.23	2007.68
27	122	4	197	0.09	2500	810000	749247.19	105.01	7135.55
28	214	4	218	0.08	2500	810000	1508911.00	111.07	13585.23
