# Supplementary material for: Human amygdala involvement in Alzheimer's disease revealed by stereological and dia‐PASEF analysis
Source: Brain Pathol. 2023 Jun 18;33(5):e13180. doi: 10.1111/bpa.13180 (PMC10467039; doi:10.1111/bpa.13180)
Supplement: Supplementary file 7 — Online Resource 7. GFAP stereological quantification data. [file BPA-33-e13180-s009.pdf]

Online Resource 7. GFAP stereological quantification data.

Table a. Cortical nucleus GFAP stereological quantification data.

	Total Markers Counted	***Number of Sections***	***Number of Sampling Sites***	***Coefficient of Error (Gundersen). m=1***	***Counting Frame Area (XY) (μm²)***	***Sampling Grid Area (XY) (μm²)***	***Estimated Population using Mean Section Thickness with Counts***	***Measured Volume (mm³)***	***Numerical Density (cell/mm³)***
AD									
1	98	4	215	0.1	2500	202500	166278.77	26.81	6202.51
2	154	4	150	0.08	2500	160000	204543.64	14.71	13901.77
3	142	4	123	0.09	2500	250000	295359.91	19.73	14969.11
4	107	3	82	0.1	2500	360000	291553.41	18.24	15983.85
5	120	4	73	0.09	2500	640000	560155.25	28.78	19461.73
6	136	4	114	0.09	2500	250000	249110.84	16.77	14856.32
7	148	4	161	0.09	2500	160000	189120.06	15.61	12114.84
8	108	3	91	0.1	2500	250000	215612.59	14.28	15096.49
9	131	4	99	0.09	2500	360000	326586.53	21.94	14886.46
10	122	4	154	0.09	2500	202500	212781.05	19.66	10824.81
Non-AD									
20	100	4	211	0.1	2500	250000	187738.31	33.70	5571.30
21	110	4	185	0.1	2500	250000	228132.63	27.92	8170.47
22	112	4	243	0.1	2500	160000	170560.41	23.66	7208.66
23	125	4	188	0.09	2500	160000	152548.56	18.83	8101.06
24	128	4	324	0.09	2500	160000	146563.95	32.26	4542.77
25	117	4	94	0.1	2500	422500	418184.72	23.24	17992.32
26	110	4	122	0.1	2500	250000	203388.19	18.78	10832.87
27	217	4	438	0.07	2500	40000	143345.16	24.65	5815.50
28	128	4	349	0.09	2500	122500	130156.94	26.79	4859.30

Table b. Basomedial nucleus GFAP stereological quantification data.

	Total Markers Counted	*Number of Sections*	*Number of Sampling Sites*	*Coefficient of Error (Gundersen). m=1*	*Counting Frame Area (XY) (μm²)*	*Sampling Grid Area (XY) (μm²)*	*Estimated Population using Mean Section Thickness with Counts*	*Measured Volume (mm³)*	*Numerical Density (cell/mm3)*
AD									
1	96	4	148	0.1	2500	422500	368178.72	40.40	9113.76
2	103	4	183	0.1	2500	90000	73934.46	9.86	7499.72
3	118	4	82	0.09	2500	250000	255008.05	12.68	20114.85
4	107	4	127	0.1	2500	360000	291439.06	30.07	9692.38
5	144	4	66	0.09	2500	640000	678869.06	26.27	25846.42
6	119	4	108	0.09	2500	160000	144979.41	10.27	14121.74
7	305	4	268	0.06	2500	62500	153734.11	10.14	15155.77
8	144	4	107	0.08	2500	202500	232144.41	13.62	17045.75
9	143	4	137	0.09	2500	202500	204493.13	16.96	12056.38
10	207	4	259	0.07	2500	90000	161628.16	14.61	11062.69
Non-AD									
19	169	4	2337	0.09	2500	10000	12862.49	14.14	909.61
20	116	4	355	0.1	2500	160000	140537.48	36.02	3902.05
21	97	4	108	0.1	2500	490000	392190.97	33.88	11577.29
22	100	4	294	0.1	2500	202500	176058.45	37.01	4757.65
23	103	4	289	0.1	2500	90000	70286.2	16.19	4342.35
24	103	4	1860	0.1	2500	15625	11638.07	17.92	649.37
25	103	4	125	0.1	2500	250000	214280.98	19.56	10952.99
26	126	4	453	0.09	2500	62500	60627.76	17.57	3450.07
27	118	4	564	0.09	2500	40000	34097.06	13.95	2444.74
28	176	4	1203	0.08	2500	22500	31177.46	16.70	1867.07

Table c. Basolateral nucleus GFAP stereological quantification data.

	Total Markers Counted	*Number of Sections*	*Number of Sampling Sites*	*Coefficient of Error (Gundersen). m=1*	*Counting Frame Area (XY) (μm²)*	*Sampling Grid Area (XY) (μm²)*	*Estimated Population using Mean Section Thickness with Counts*	*Measured Volume (mm³)*	*Numerical Density (cell/mm³)*
AD									
1	111	4	221	0.1	2500	562500	539383.38	79.12	6817.54
2	109	4	147	0.1	2500	490000	424489.09	44.14	9617.03
3	135	4	125	0.09	2500	640000	744446.81	50.16	14842.27
4	145	4	316	0.08	2500	490000	531520	98.90	5374.14
5	138	4	99	0.09	2500	1210000	1260234.75	73.97	17036.92
6	165	4	200	0.08	2500	250000	313094.78	30.55	10250.21
7	150	4	142	0.09	2500	562500	678391.69	50.76	13365.45
8	140	4	130	0.09	2500	640000	715452.25	54.26	13185.53
9	140	4	161	0.09	2500	640000	639029.19	64.91	9844.85
10	127	4	162	0.09	2500	490000	549225	51.10	10748.67
Non-AD									
19	104	4	729	0.1	2500	160000	143050.55	74.29	1925.50
20	109	4	420	0.1	2500	360000	299752.56	95.92	3124.96
21	119	4	121	0.1	2500	810000	777483.19	61.49	12643.59
22	113	4	275	0.1	2500	360000	404893.06	63.21	6405.49
23	101	4	245	0.1	2500	490000	386933.78	76.26	5074,10
24	135	4	4293	0.1	2500	22500	23010.16	60.92	377.68
25	104	4	177	0.1	2500	490000	440181.28	53.58	8214.78
26	113	4	158	0.1	2500	640000	566990.13	65.20	8696.55
27	129	4	374	0.09	2500	250000	251255.75	58.14	4321.57
28	149	4	1230	0.09	2500	90000	108668.56	69.83	1556.22

Table d. Lateral nucleus GFAP stereological quantification data.

	Total Markers Counted	*Number of Sections*	*Number of Sampling Sites*	*Coefficient of Error (Gundersen). m=1*	*Counting Frame Area (XY) (μm²)*	*Sampling Grid Area (XY) (μm²)*	*Estimated Population using Mean Section Thickness with Counts*	*Measured Volume (mm³)*	*Numerical Density (cell/mm³)*
AD									
1	190	4	239	0.08	2500	250000	717146.19	64.62	11098.50
2	152	4	199	0.09	2500	810000	766359.81	81.76	9373.65
3	94	4	91	0.1	2500	810000	839913.06	57.90	14506.87
4	124	4	142	0.09	2500	490000	1110507.38	112.42	9877.94
5	98	4	58	0.1	2500	640000	1574430.25	87.45	18004.68
6	239	4	340	0.07	2500	160000	865646.44	105.03	8241.90
7	198	4	177	0.08	2500	810000	1596515.63	114.52	13940.45
8	142	4	141	0.09	2500	1440000	1155604.5	92.27	12524.60
9	163	4	223	0.09	2500	490000	660617.06	79.20	8340.98
10	207	4	259	0.08	2500	360000	814232.06	92.48	8804.87
Non-AD									
19	105	4	841	0.1	2500	422500	233614.55	135.05	1729.89
20	103	4	291	0.1	2500	640000	654654.38	154.38	4240.62
21	134	4	164	0.09	2500	1000000	858425.63	82.56	10398.08
22	137	4	293	0.09	2500	1210000	639179.5	90.24	7083.02
23	147	4	300	0.08	2500	2250000	759134.63	124.42	6101.19
24	126	4	1364	0.09	2500	490000	160849.98	138.70	1159.72
25	117	4	203	0.09	2500	1000000	797201.56	108.14	7372.08
26	138	4	170	0.09	2500	1000000	1563261	154.95	10088.68
27	151	4	300	0.08	2500	562500	577616.94	95.60	6042.20
28	111	4	476	0.1	2500	640000	328507.84	109.44	3001.74
