# Supplementary material for: Human amygdala involvement in Alzheimer's disease revealed by stereological and dia‐PASEF analysis
Source: Brain Pathol. 2023 Jun 18;33(5):e13180. doi: 10.1111/bpa.13180 (PMC10467039; doi:10.1111/bpa.13180)
Supplement: Supplementary file 8 — Online Resource 8. Aβ stereological quantification data. [file BPA-33-e13180-s010.pdf]

Online Resource 8. Aβ stereological quantification data.

Table a. Cortical nucleus Aβ stereological quantification data.

	Marker Count	*Number of Sections*	*Number of Sampling Sites*	*Coefficient of Error (Gundersen). m=1*	*Counting Frame Area (XY) (μm²)*	*Sampling Grid Area (XY) (μm²)*	*Grid Spacing (μm)*	*Area Fraction*
AD								
1	141	4	95	0.027	40000	490000	40	0.0717
2	65	3	17	0.041	40000	1562500	40	0.1857
3	60	4	21	0.044	40000	2250000	40	0.1245
4	65	3	23	0.064	40000	1440000	40	0.1474
5	53	4	26	0.044	40000	2250000	40	0.0971
6	45	3	13	0.051	40000	2250000	40	0.1385
7	54	4	32	0.053	40000	1000000	40	0.0897
8	19	4	17	0.095	40000	2250000	40	0.0554
9	43	3	13	0.058	40000	2250000	40	0.1552
10	79	4	38	0.043	40000	1000000	40	0.1006

Table b. Basomedial nucleus Aβ stereological quantification data.

	Marker Count	*Number of Sections*	*Number of Sampling Sites*	*Coefficient of Error (Gundersen). m=1*	*Counting Frame Area (XY) (μm²)*	*Sampling Grid Area (XY) (μm²)*	*Grid Spacing (μm)*	*Area Fraction*
AD								
1	85	4	66	0.036	40000	1000000	40	0.0587
2	95	4	29	0.03	40000	810000	40	0.1457
3	27	4	14	0.066	40000	2250000	40	0.0888
4	52	3	18	0.062	40000	2250000	40	0.1354
5	51	4	22	0.043	40000	2250000	40	0.1028
6	19	4	8	0.098	40000	2250000	40	0.125
7	39	4	19	0.062	40000	1000000	40	0.097
8	27	4	13	0.087	40000	2250000	40	0.0982
9	29	4	12	0.063	40000	2250000	40	0.101
10	67	4	31	0.041	40000	1000000	40	0.1012

Table c. Basolateral nucleus Aβ stereological quantification data.

	Marker Count	*Number of Sections*	*Number of Sampling Sites*	*Coefficient of Error (Gundersen). m=1*	*Counting Frame Area (XY) (μm²)*	*Sampling Grid Area (XY) (μm²)*	*Grid Spacing (μm)*	*Area Fraction*
AD								
1	51	4	92	0.055	40000	2250000	40	0.025
2	96	4	54	0.039	40000	2250000	40	0.0789
3	61	4	24	0.046	40000	4000000	40	0.1068
4	40	3	19	0.064	40000	6250000	40	0.0855
5	85	4	46	0.037	40000	3062500	40	0.0819
6	34	4	15	0.078	40000	4000000	40	0.1073
7	28	4	20	0.072	40000	4000000	40	0.061
8	34	4	14	0.068	40000	6250000	40	0.1009
9	42	4	19	0.063	40000	6250000	40	0.0995
10	87	4	46	0.053	40000	2250000	40	0.0921

Table d. Lateral nucleus Aβ stereological quantification data.

	Marker Count	***Number of Sections***	***Number of Sampling Sites***	***Coefficient of Error (Gundersen). m=1***	***Counting Frame Area (XY) (µm²)***	***Sampling Grid Area (XY) (µm²)***	***Grid spacing (µm)***	***Area Fraction***
AD								
1	204	4	139	0.026	40000	810000	40	0.0639
2	80	4	58	0.045	40000	2250000	40	0.0634
3	36	4	27	0.057	40000	4000000	40	0.0551
4	26	3	24	0.079	40000	6250000	40	0.0538
5	38	4	37	0.062	40000	4000000	40	0.0431
6	49	4	30	0.049	40000	6250000	40	0.0725
7	38	4	21	0.063	40000	9000000	40	0.0792
8	41	4	16	0.053	40000	9000000	40	0.1111
9	39	4	22	0.096	40000	6250000	40	0.0901
10	41	4	40	0.054	40000	4000000	40	0.0422
