# Supplementary material for: Human amygdala involvement in Alzheimer's disease revealed by stereological and dia‐PASEF analysis
Source: Brain Pathol. 2023 Jun 18;33(5):e13180. doi: 10.1111/bpa.13180 (PMC10467039; doi:10.1111/bpa.13180)
Supplement: Supplementary file 9 — Online Resource 9. Tau stereological quantification data. [file BPA-33-e13180-s007.pdf]

Online Resource 9. Tau stereological quantification data.

Table a. Cortical nucleus Tau stereological quantification data.

	Marker Count	***Number of Sections***	***Number of Sampling Sites***	***Coefficient of Error (Gundersen). m=1***	***Counting Frame Area (XY) (μm²)***	***Sampling Grid Area (XY) (μm²)***	***Grid Spacing (μm)***	***Area Fraction***
AD								
1	40	4	11	0.06	10000	4000000	20	0.1695
2	47	3	11	0.056	10000	2250000	20	0.1992
3	50	4	9	0.047	10000	4000000	20	0.2315
4	46	3	7	0.049	10000	4000000	20	0.2805
5	68	4	8	0.04	10000	9000000	20	0.34
6	45	3	6	0.048	10000	4000000	20	0.3
7	67	4	14	0.045	10000	2250000	20	0.2134
8	36	4	6	0.069	10000	4000000	20	0.3243
9	67	3	14	0.064	10000	4000000	20	0.3028
10	74	4	11	0.038	10000	4000000	20	0.3122

Table b. Basomedial nucleus Tau stereological quantification data.

	Marker Count	*Number of Sections*	*Number of Sampling Sites*	*Coefficient of Error (Gundersen). m=1*	*Counting Frame Area (XY) (µm²)*	*Sampling Grid Area (XY) (µm²)*	*Grid Spacing (µm)*	*Area Fraction*
AD								
1	64	4	11	0.038	10000	6250000	20	0.2327
2	67	4	13	0.055	10000	1440000	20	0.2094
3	43	4	8	0.053	10000	2250000	20	0.215
4	45	3	7	0.066	10000	6250000	20	0.2903
5	25	4	7	0.041	10000	9000000	20	0.1667
6	43	4	7	0.073	10000	2250000	20	0.3071
7	71	4	18	0.035	10000	1000000	20	0.1578
8	23	4	4	0.086	10000	6250000	20	0.2614
9	71	4	18	0.065	10000	4000000	20	0.1799
10	35	4	6	0.062	10000	9000000	20	0.2333

Table c. Basolateral nucleus Tau stereological quantification data.

	Marker Count	*Number of Sections*	*Number of Sampling Sites*	*Coefficient of Error (Gundersen). m=1*	*Counting Frame Area (XY) (μm²)*	*Sampling Grid Area (XY) (μm²)*	*Grid Spacing (μm)*	*Area Fraction*
AD								
1	44	4	17	0.056	10000	6250000	20	0.1183
2	81	4	13	0.035	10000	6250000	20	0.2492
3	72	4	17	0.038	10000	6250000	20	0.1946
4	50	3	15	0.054	10000	9000000	20	0.1618
5	79	4	17	0.088	10000	6250000	20	0.238
6	52	4	6	0.047	10000	9000000	20	0.3467
7	47	4	12	0.051	10000	6250000	20	0.1649
8	54	4	8	0.046	10000	9000000	20	0.27
9	47	4	12	0.038	10000	9000000	20	0.2527
10	55	4	9	0.062	10000	9000000	20	0.2444

Table d. Lateral nucleus Tau stereological quantification data.

	Marker Count	*Number of Sections*	*Number of Sampling Sites*	*Coefficient of Error (Gundersen). m=1*	*Counting Frame Area (XY) (µm²)*	*Sampling Grid Area (XY) (µm²)*	*Grid spacing (µm)*	*Area Fraction*
AD								
1	86	4	19	0.037	10000	4000000	20	0.1811
2	36	4	17	0.058	10000	9000000	20	0.0891
3	39	4	16	0.055	10000	6250000	20	0.1037
4	76	3	16	0.038	10000	9000000	20	0.1995
5	61	4	13	0.037	10000	6250000	20	0.1877
6	71	4	22	0.035	10000	9000000	20	0.1312
7	28	4	14	0.079	10000	12250000	20	0.08
8	80	4	24	0.034	10000	6250000	20	0.1382
9	28	4	14	0.059	10000	9000000	20	0.095
10	33	4	12	0.06	10000	12250000	20	0.11
