# Supplementary material for: Human amygdala involvement in Alzheimer's disease revealed by stereological and dia‐PASEF analysis
Source: Brain Pathol. 2023 Jun 18;33(5):e13180. doi: 10.1111/bpa.13180 (PMC10467039; doi:10.1111/bpa.13180)
Supplement: Supplementary file 10 — Online Resource 10. Phosphorylated TDP‐43 (TDP‐43‐P) deposits in amygdala in AD. (a) Immunohistochemistry against TDP‐43‐P in AC in AD (case 2). Note different deposition pattern between nuclei. (b) Abundant cellular accumulations of TDP‐43‐P can be observed in Co, while in (c) BM and (d) BLTDP‐43‐P appears in clusters. In contrast, (e) scarce deposits are in La. Co: Cortical nucleus, BM: Basomedial nucleus, BL: Basolateral nucleus, La: Lateral nucleus, EC: Entorhinal cortex. Scale bar = 1000 μm in (a); and 50 μm in (b,c,d,e). [file BPA-33-e13180-s002.pdf]

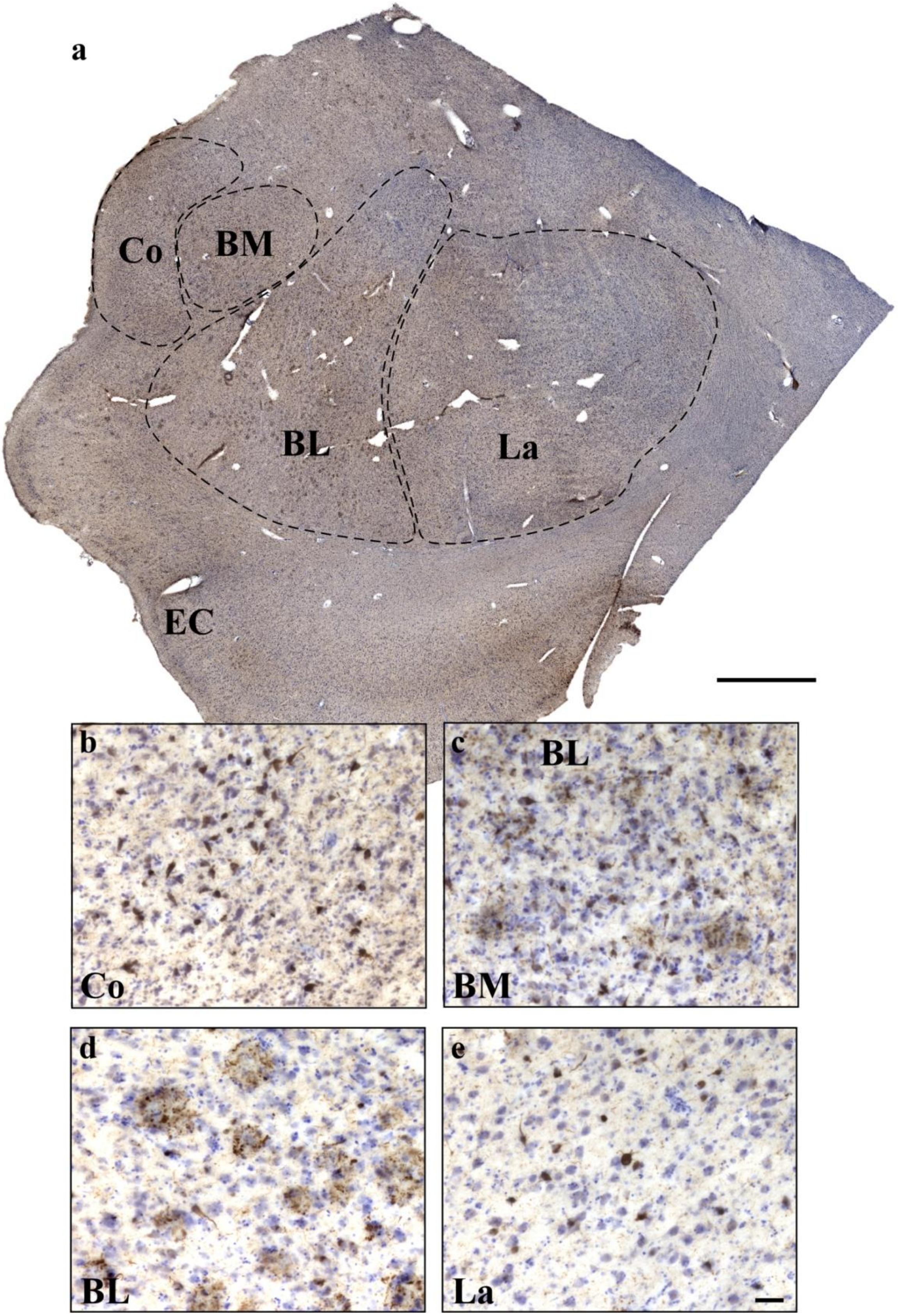


**Online Resource 10** *Phosphorylated TDP-43 (TDP-43-P) deposits in amygdala in AD*. (a) Immunohistochemistry against TDP-43-P in AC in AD (case 2). Note different deposition pattern between nuclei. (b) Abundant cellular accumulations of TDP-43-P can be observed in Co, while in (c) BM and (d) BL TDP-43-P appears in clusters. In contrast, (e) scarce deposits are in La. Co: Cortical nucleus, BM: Basomedial nucleus, BL: Basolateral nucleus, La: Lateral nucleus, EC: Entorhinal cortex. Scale bar = 1000 µm in (a); and 50 µm in (b, c, d, e)
