# Supplementary material for: Human amygdala involvement in Alzheimer's disease revealed by stereological and dia‐PASEF analysis
Source: Brain Pathol. 2023 Jun 18;33(5):e13180. doi: 10.1111/bpa.13180 (PMC10467039; doi:10.1111/bpa.13180)
Supplement: Supplementary file 12 — Online Resource 12. Proteomic analysis. Volcano plot (a) showed 108 up‐(green) and 70 downregulated (red) DEPs (FC > 1.5, p value <0.05). Venn diagrams identified that are present in the different cellular types, neurons (b), microglia (c) and astrocytes (d), and interact with pathological proteinsin AD. Cellular responses to stress, regulation of proteolysis and response to wounding were exposed as impaired processes by functional analysis with Metascape (f). DEPs: differentially expressed proteins. [file BPA-33-e13180-s011.pdf]

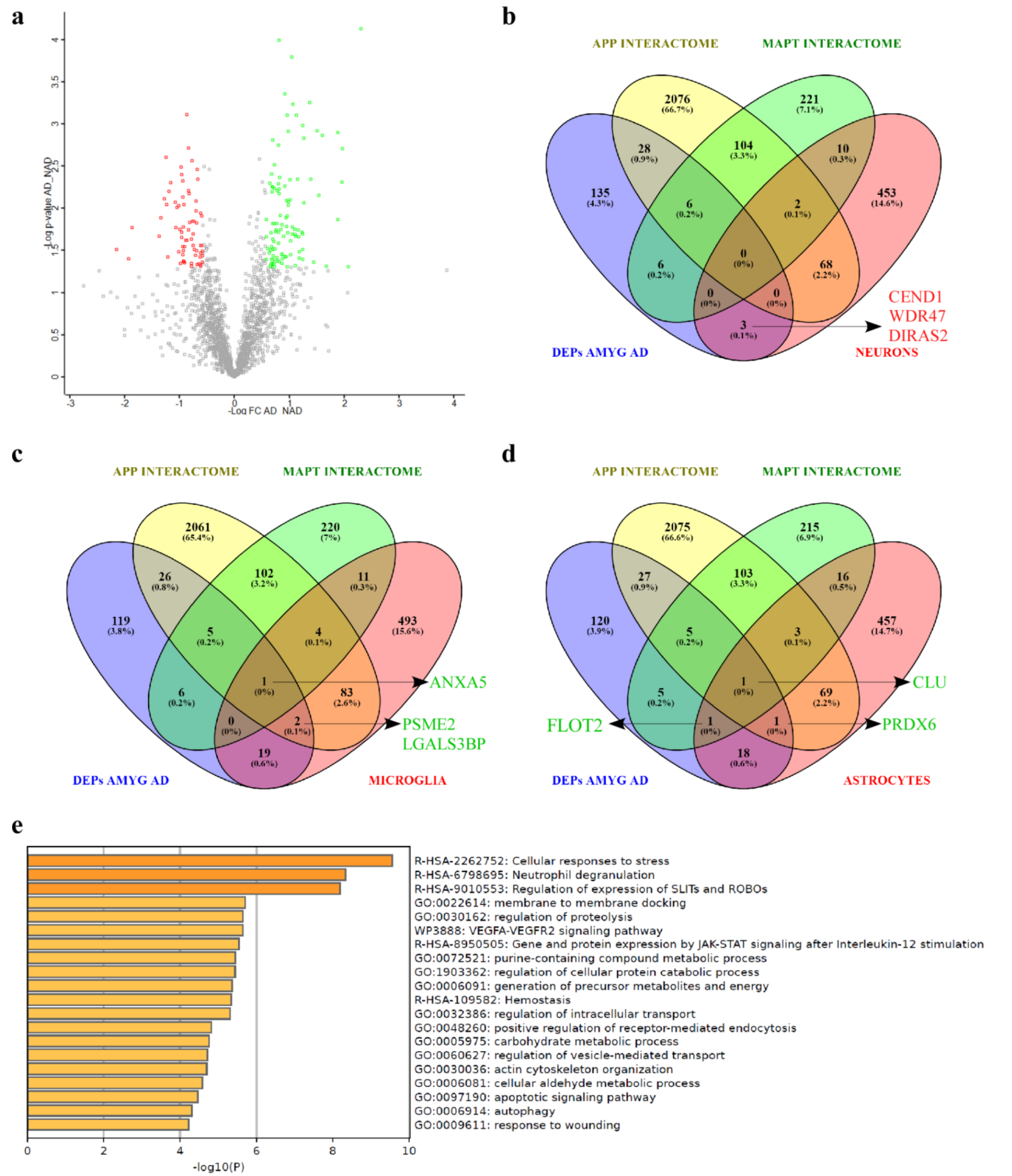

Online Resource 12 *Proteomic analysis*. Volcano plot (a) showed 108 up- (green) and 70 downregulated (red) DEPs (FC > 1.5, p value < 0.05). Venn diagrams identified that are present in the different cellular types, neurons (b), microglia (c) and astrocytes (d), and interact with pathological proteins in AD. Cellular responses to stress, regulation of proteolysis and response to wounding were exposed as impaired processes by functional analysis with Metascape (f). DEPs: differentially expressed proteins
